# An engineered palivizumab IgG2 subclass for synthetic gp130 and fas-mediated signaling

**DOI:** 10.1016/j.jbc.2025.108205

**Published:** 2025-01-17

**Authors:** Christoph Wittich, Julia Ettich, Marcel Hertell, Biswadeep Ghosh Roy, Haifeng C. Xu, Doreen M. Floss, Philipp A. Lang, Jürgen Scheller

**Affiliations:** 1 Institute of Biochemistry and Molecular Biology II, Medical Faculty and University Hospital Düsseldorf, Heinrich Heine University, Düsseldorf, Germany; 2 Institue of Molecular Medicine II, Medical Faculty and University Hospital Düsseldorf, Heinrich Heine University, Düsseldorf, Germany

**Keywords:** synthetic cytokine, palivizumab, interleukin 6, Fas, apoptosis, scFv, gp130

## Abstract

Recently, we phenocopied interleukin (IL-)6 signaling using the dimerized single-chain variable fragment (scFv) derived from the respiratory syncytial virus IgG1-antibody palivizumab (P^scFv^LHFc) to activate a palivizumab antiidiotypic nanobody (AIP^VHH^)-gp130 receptor fusion protein. Palivizumab was unable to activate STAT3 signaling, so we aimed to create a similar ligand capable of triggering this pathway. Here, we created three variants of the ligand called P^scFv^LH0Fc, P^scFv^LH4Fc and P^scFv^LH8Fc by shortening the spacer region connecting P^scFv^LH and Fc from 23 amino acids in P^scFv^LHFc to 0 amino acids or expanding it by rigid linkers of four or eight alpha helical loops, respectively. The rigid-linker ligands had completely altered cellular activation patterns *via* AIP^VHH^gp130 fusion proteins. Deleting the extracellular stalk region between transmembrane and AIP^VHH^ in the synthetic receptors AIP2^VHH^gp130Δstalk and AIP3^VHH^gp130Δstalk to increase rigidity and enhanced the biological activity of the short spacer P^scFv^Fc ligands. Since scFv constructs are less stable than antibodies and have not been Food and Drug Administration approved, we looked for different antibody backbones. Transferring palivizumab's variable region to a more rigid and hence more agonistic IgG2 backbone (P^IgG2^) maintained affinity while improving agonistic properties activating cells expressing AIP2^VHH^gp130Δstalk and AIP3^VHH^gp130Δstalk but not their full-length counterparts. Furthermore, we engineered a tetravalent palivizumab variant (P^scFv^P^IgG2^) capable of inducing higher-order receptor clustering, activating Fas-induced apoptosis. In summary, we engineered a fully-synthetic cytokine/cytokine receptor pair based on the IgG2-variant of palivizumab and the AIP^VHH^gp130Δstalk variants opening avenues for therapeutic applications using nonphysiological targets in immunotherapy.

Upon cytokine binding, cytokine-receptors undergo a transition from a monomeric inactive state to a dimeric or multimeric active state (1). Recent developments in synthetic biology have introduced antibodies as cytokine modulators, representing a promising class of therapeutic biomolecules (2, 3). Additionally, CAR T-cell therapy, an approved treatment for severe acute lymphatic leukemia (4), exemplifies the successful application of synthetic receptors using antibody fragments for tumor antigen recognition (5).

We have developed a first generation synthetic cytokine receptor (SyCyR^1st^) system, comprising nanobody-based (6) cytokine receptors and soluble protein dimers that serve as ligands (7). This system originally uses nanobodies recognizing GFP or mCherry (8, 9). These nanobodies were derived from Camelidae heavy chain antibodies, which are known for their immunological safety and therapeutic potential (10), this is however not true for the dimeric GFP/mCherry ligands which might provoke antibody responses after repeated *in vivo* applications (11).

To enhance the system's applicability and achieve the objective of generating a ligand–receptor pair capable of activating STAT3 signaling, we designed second-generation SyCyRs (SyCyR^2nd^) using nonimmunogenic synthetic ligands based on antibody–antiidiotypic nanobody pairs (12, 13). In this configuration, the antiidiotypic nanobody is fused to the transmembrane and intracellular domain of the receptor of interest. The development of these tailor-made SyCyR systems presents exciting possibilities for enhancing CAR T-cell therapies (14). By engineering receptors capable of modulating CAR T-cell activity, we might provide a valuable tool for fine-tuning cellular responses like enhancing T-cell activation (15) or overcoming T-cell exhaustion (16). A critical factor in this modulation is the role of juxtamembrane amino acids of the synthetic receptor connecting the transmembrane and the extracellular domain, which significantly influence the signaling activity of gp130 (17) and other synthetic receptor systems, such as generalized extracellular molecule sensors (18). Therefore, we engineered the short extracellular stalk region in SyCyR^2nd^ originally taken from native gp130. By shortening this region, we aimed to increase interaction rigidity and introduce a different rotation to the cytoplasmic side of receptors in third generation SyCyRs (SyCyR^3rd^).

As a ligand for the second and third generation SyCyRs we used variants of palivizumab (19), a humanized monoclonal antibody targeting the respiratory syncytial virus (20) and approved for human use, as nanobody-cytokine receptor pair. This approach enables background-free, cell-type-specific activation of SyCyRs, since palivizumab has no targets in humans and hence only engineered cells are affected. Our SyCyR fusion protein (AIP^VHH^gp130) can be activated by a reformatted dimeric P^scFv^Fc. Here, the variable domains of palivizumab were engineered as single-chain Fv fragment with sustained binding capacity and specificity, and directly fused to an immunoglobulin (Ig)G1 Fc part to achieve ligand dimerization. Albeit P^scFv^Fc was biologically active, this ligand format has limited therapeutic potential because many such fusion proteins are only stable for a few hours or days after their experimental incorporation into human serum (21) and none have been approved to date. It is worth noting that attempts to activate SyCyR^2nd^ using original palivizumab IgG1 were unsuccessful, underscoring the system's complexity and the need for further optimization (12). To engineer a functional SyCyR:palivizumab pair for therapeutic settings, it is therefore crucial to use an antibody format that could be licensed for human applications. Given our initial unsuccessful attempt using the original palivizumab IgG1, we now focused on reengineering the receptor and ligand components to achieve functionality while maintaining suitability for clinical applications. Engineered antibodies exhibit diverse functional characteristics, including antagonistic or agonistic properties. The structural rigidity of the hinge region plays a crucial role in determining an antibody's agonistic potential, with more rigid hinge regions typically displaying enhanced agonistic activity (22). This relationship between structural rigidity and functional properties offers opportunities for engineering antibodies with tailored activities for specific applications. To improve palivizumab's functionality as a dimerizer for AIP^VHH^gp130, we opted for an IgG2 subclass backbone with a rigid hinge region (23). We transferred the variable regions from palivizumab (P^IgG1^) onto this engineered IgG2 backbone, creating P^IgG2^.

Taken together, the combination of IgG2-reformatted palivizumab and stalk shortening in SyCyR^3rd^ resulted in effective activation, opening promising opportunities for therapeutic applications using engineered antibodies like P^IgG2^ to activate SyCyRs on Ba/F3 and primary T-cells.

## Results

### Engineering stalk domain deletion variants of the synthetic AIP^VHH^gp130 receptors

**Figure 1:**
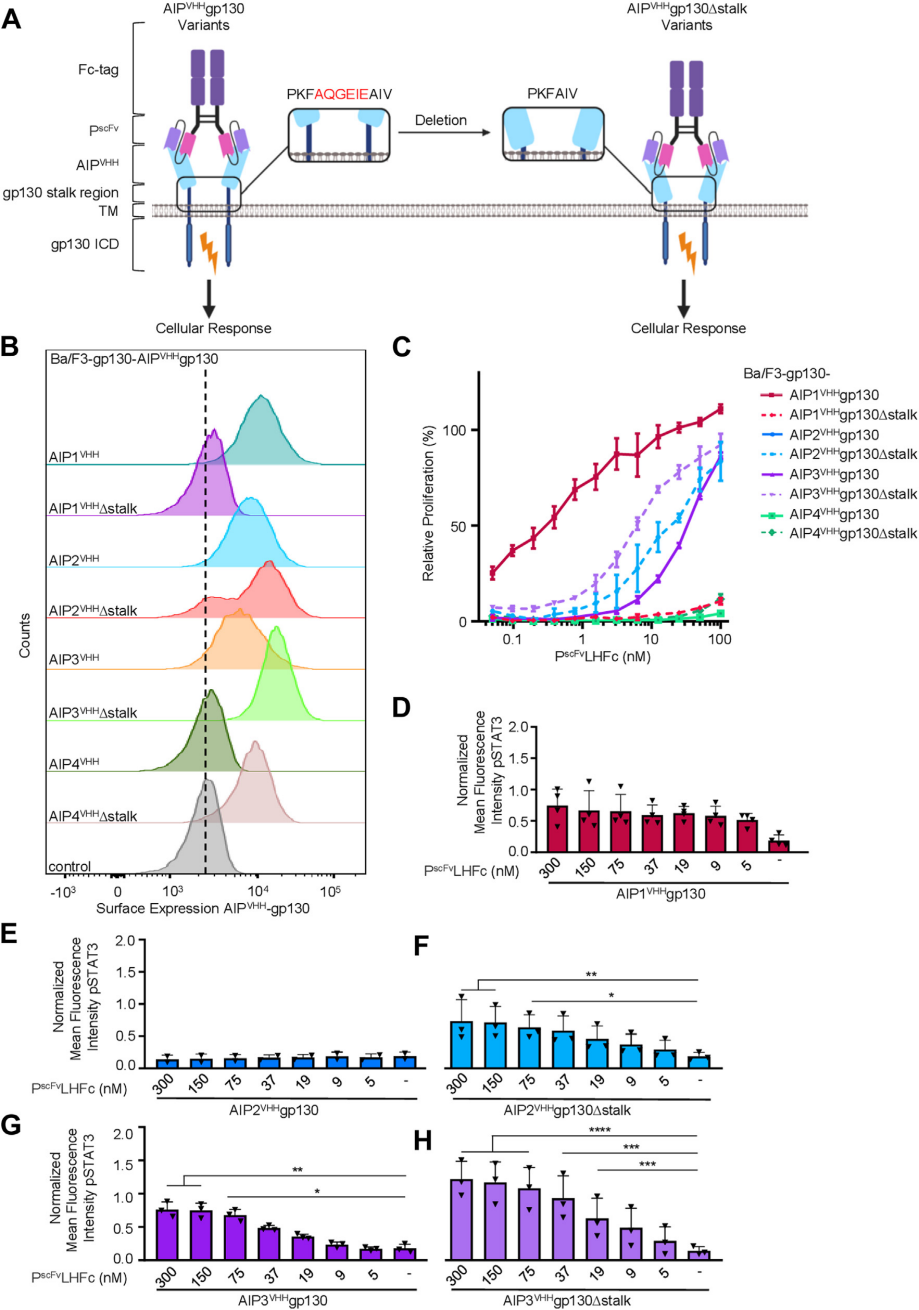
**P**^**scFvLH23**^**Fc activates AIP**^**VHH**^**gp130 different than AIP**^**VHH**^**gp130Δstalk**. *A*, schematic illustration of the transformation from AIP^VHH^gp130 to AIP^VHH^gp130Δstalk. *B*, flow cytometric analysis of myc-tagged synthetic receptors on Ba/F3-gp130 cells expressing AIP1-4^VHH^gp130 and AIP1-4^VHH^gp130Δstalk. *C*, dose-dependent proliferation of Ba/F3-gp130 cells expressing AIP1-4^VHH^gp130 and AIP1-4^VHH^gp130 Δstalk in response to PscFvLH23Fc (0.05–300 nM). Proliferation was normalized to HIL-6 (10 ng/ml) induced proliferation for each cell line. Data represent mean ± S.D. of three biological replicates from one representative experiment out of three. *D*–*F*, normalized mean fluorescence intensity of STAT3 phosphorylation in response to P^scFv^LHFc (5–300 nM) for (*D*) AIP1^VHH^gp130, (*E*) AIP2^VHH^gp130, (*F*) AIP2^VHH^gp130Δstalk, (*G*) AIP3^VHH^gp130, and (*H*) AIP3^VHH^gp130Δstalk. Data represent mean ± S.D. of three biological replicates (n = 3). HIL-6, hyper-IL-6; scFV, single-chain variable fragment.

Previously, we described the generation of four antiidiotypic nanobodies (VHHs) binding to the respiratory syncytial virus IgG1-monoclonal antibody palivizumab with high affinity (12). AIP1-4^VHH^ was genetically fused to a complementary DNA coding for the transmembrane and intracellular domain of gp130 and named AIP1^VHH^gp130, AIP2^VHH^gp130, AIP3^VHH^gp130, and AIP4^VHH^gp130 (Fig. 1*A*). Notably, only AIP1^VHH^gp130 and AIP3^VHH^gp130 conferred signaling after stimulation with a dimerized single-chain variable fragment (scFv) derived from palivizumab (P^scFv^LHFc), whereas full-length palivizumab failed to activate signaling *via* any of the four AIP^VHH^gp130 synthetic receptors (12). The architecture of the AIP1-4^VHH^gp130 receptors, included a six amino acid residue long stalk region (AQGEIE) taken from gp130 to separate the AIP^VHH^ from the transmembrane domain and allow some flexibility in the positioning of AIP^VHH^. To analyze the effect on signaling of directly positioning the AIP^VHH^ on the membrane, we deleted the complete stalk region in AIP1-4^VHH^gp130Δstalk variants (Fig. 1*A*). Expression of AIP^VHH^gp130Δstalk proteins on the cell surface of transduced Ba/F3-gp130 cells was shown by flow cytometry against the N-terminal myc-tag (Fig. 1*B*) and revealed that deletion of the stalk region resulted in higher expression levels compared to the original AIP2-4^VHH^gp130 variants, but not for AIP1^VHH^gp130. This might be of importance since the expression levels could influence the concentrations needed for cellular activation and might contribute to the distinct behavior of the receptors on the cells. Ba/F3-gp130 cells are murine pre-B cells, and due to stable expression of gp130, these cells become responsive to hyper-IL-6 (HIL-6, fusion protein of interleukin (IL)-6 and soluble IL-6 receptor α), which induces cell proliferation *via* JAK/STAT signaling (24). After the introduction of AIP^VHH^gp130 into Ba/F3-gp130 cells, the proliferation of these cells became dependent on the synthetic ligand P^scFv^LHFc (12) (schematic Fig. 1*A*, experiment Fig. 1*C*). Interestingly, deletion of the stalk region in the AIP1-4^VHH^gp130Δstalk rendered the previously inactive synthetic receptor AIP2^VHH^gp130 into an active state in stably transduced Ba/F3-gp130 cells. As observed previously, P^scFv^LHFc activated AIP1^VHH^gp130 (EC50 = 0.15 nM) and AIP3^VHH^gp130 (EC50 = 39.07 nM) in Ba/F3-gp130 cells, but also activation of AIP2^VHH^gp130Δstalk (EC50 = 18.31 nM) and AIP3^VHH^gp130Δstalk (EC50 = 6.07 nM) in Ba/F3-gp130 cells was observed. Whereas AIP2^VHH^gp130, AIP4^VHH^gp130, AIP1^VHH^gp130Δstalk, and AIP4^VHH^gp130Δstalk were not active after stimulation with P^scFv^LHFc (Fig. 1*C*). Subsequently, the same activation pattern was observed for phosphorylated STAT3 of SyCyR expressing Ba/F3-gp130 cells after 45 min stimulation with P^scFv^LHFc (Figs. 1, *D*–*H* and S1). Another downstream signal of gp130, STAT1 (25) was also phosphorylated in both AIP2gp130Δstalk and AIP3gp130Δstalk (Fig. S2). In conclusion, our data showed that editing of AIP-SyCyRs by introducing STALK deletions resulted in the activation of three (AIP1,2,3^VHH^) instead of only two (AIP1,3^VHH^) of the four originally described synthetic AIP^VHH^gp130 cytokine receptors.

### Decreasing the linker peptide length in P^scFv^LHFc sharpened biological activity

**Figure 2:**
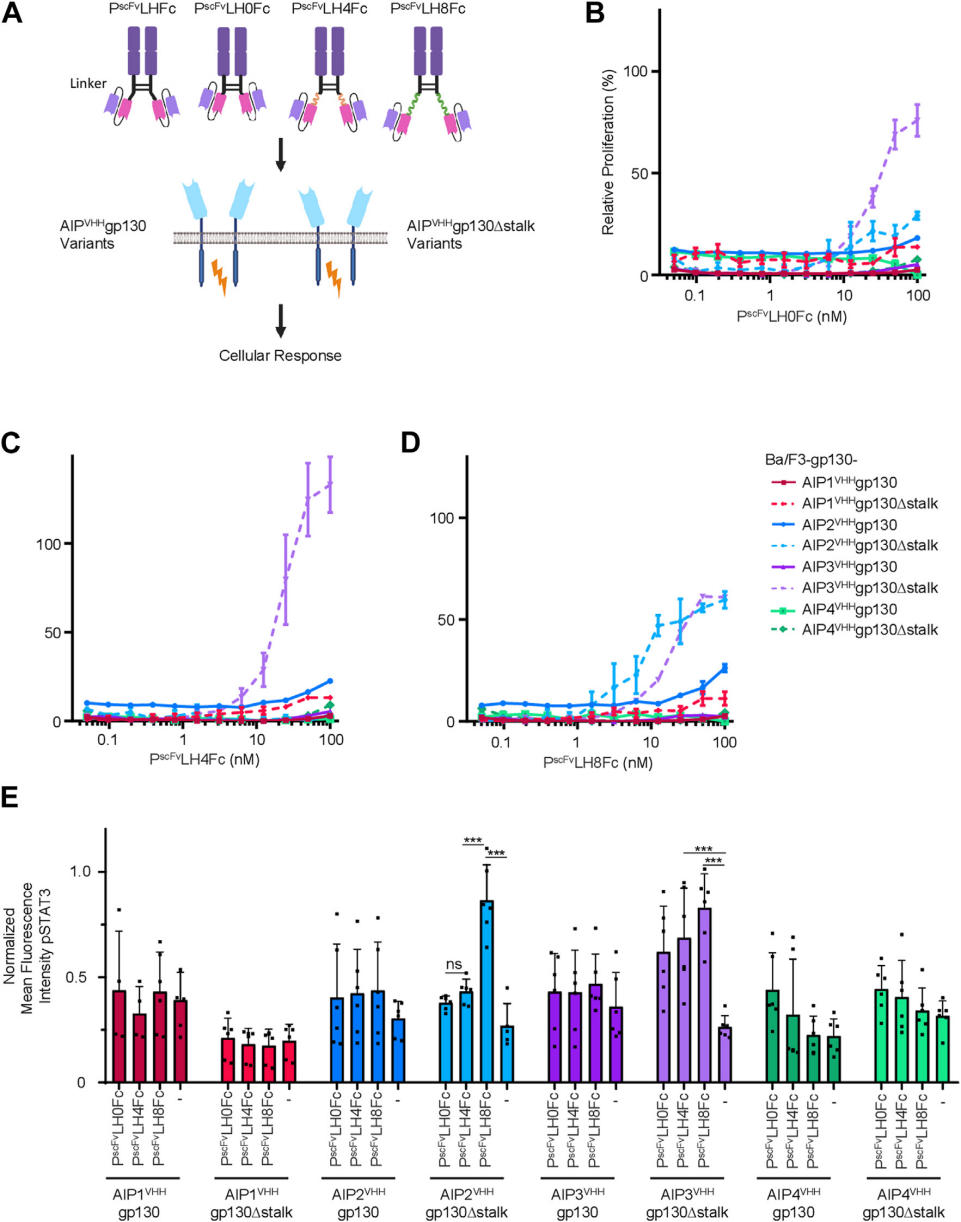
**P**^**scFvLH**^**Fc of varying size differentially activate AIP**^**VHH**^**gp130 variants.***A*, schematic illustration of P^scFv^ with different linker lengths and all SyCyR variants. *B*–*D*, dose-dependent proliferation of Ba/F3-gp130 cells expressing AIP1-4^VHH^gp130 and AIP1-4^VHH^gp130stalk in response to (*B*) P^scFv^LH0Fc, (*C*) P^scFv^LH4Fc, and (*D*) P^scFv^LH8Fc (0.05–300 nM). Proliferation was normalized to HIL-6 (10 ng/ml) induced proliferation for each cell line. Data represent mean ± S.D. of three biological replicates from one representative experiment out of three. *E*, normalized mean fluorescence intensity of STAT3 phosphorylation in response to P^scFv^LH0Fc, P^scFv^LH4Fc, and P^scFv^LH8Fc (300 nM) for all AIP^VHH^gp130 and AIP^VHH^gp130Δstalk variants. Data represent mean ± S.D. of three biological replicates (n = 3). HIL-6, hyper-IL-6; scFV, single-chain variable fragment; SyCyR, synthetic cytokine receptor.

In P^scFv^LHFc, the single-chain Fv fragments with the combination of the variable domains of the light chain at the N terminus and the heavy chain at the C terminus was fused by a flexible peptide linker of 23 amino acid residues to an Fc part of an IgG1 antibody (26) (Fig. 2*A*). To reduce the linker length flexibility, we derived P^scFv^LH0Fc by deleting all 23 linker residues from P^scFv^LHFc. Subsequently we inserted four and eight EAAAK repeats between the scFv and the Fc-tag, which form rigid alpha-helical loops resulting in P^scFv^LH4Fc and P^scFv^LH8Fc, respectively (Fig. 2*A*, complete sequences in Fig. S3). P^scFv^LH0Fc, P^scFv^LH4Fc, and P^scFv^LH8Fc were expressed in Expi293F cells and purified as dimers from the cell supernatants *via* Protein A affinity chromatography (Fig. S4, *A* and *B*). Next, the affinities of captured P^scFv^LH0Fc, P^scFv^LH4Fc, and P^scFv^LH8Fc to soluble AIP2^VHH^ were determined by surface plasmon resonance. AIP2^VHH^ binds P^scFv^LH0Fc, P^scFv^LH4Fc, and P^scFv^LH8Fc with comparable affinities of 20 nM, 14 nM, and 12 nM, respectively (Fig. S5, *A*–*C*), which is in good agreement with the previously determined KD of 2.2 nM for AIP2^VHH^ to palivizumab (12). Next, we tested P^scFv^LH0Fc, P^scFv^LH4Fc, and P^scFv^LH8Fc on Ba/F3-gp130 cells expressing any of the AIP1-4^VHH^gp130 or AIP1-4^VHH^gp130Δstalk variants for their ability to induce cellular proliferation and STAT3 phosphorylation (schematic Fig. 2*A*). Dose-dependent stimulation of Ba/F3-gp130 cells expressing AIP^VHH^gp130 variants with P^scFv^LH0Fc, P^scFv^LH4Fc, and P^scFv^LH8Fc revealed no induction of cellular proliferation (Fig. 2, *B*–*D*, legend in 2B). P^scFv^LH0Fc, P^scFv^LH4Fc, and P^scFv^LH8Fc also failed to induce sustained STAT3 phosphorylation in these cells, as determined by flow cytometry using antibodies directed against phosphorylated and nonphosphorylated STAT3 (Figs. 2*E* and S6). Of note, stimulation with the synthetic ligands P^scFv^LH0Fc, P^scFv^LH4Fc, and P^scFv^LH8Fc of Ba/F3-gp130 cells expressing the AIP^VHH^gp130Δstalk variants showed that all ligands induced cellular proliferation *via* AIP3^VHH^gp130Δstalk with EC50s of 26.31 nM, 22.05 nM, and 17.63 nM and P^scFv^LH8Fc also activated *via* AIP2^VHH^gp130Δstalk with an EC50 of 6.954 nM (Fig. 2, *B*–*D*). Significantly increased STAT3 phosphorylation was detected for stimulation of AIP3^VHH^gp130Δstalk with P^scFv^LH4Fc, as well as AIP2^VHH^gp130Δstalk, and AIP3^VHH^gp130Δstalk with P^scFv^LH8Fc (Fig. 2*E*). Our data demonstrated that shortening of the linker length between the scFv and N-terminal cysteine of the Fc hinge region shifted the biological activity of the synthetic ligands toward different receptor compositions suggesting that receptor activation capacity is influenced on both sides, the ligand and the receptor. Moreover, the original linker length of 23 amino acid residues in P^scFv^LHFc appears to be optimal for achieving the broadest activation spectrum of this SyCyR system. While our experimental data did not reveal a systematic activation pattern, and current computational approaches, including AI-driven protein structure prediction, also face significant limitations when modeling complex membrane receptor systems. Although these tools can provide valuable insights for initial design strategies, the development of functional receptor-ligand pairs still requires empirical optimization through systematic experimental testing.

### An engineered IgG2 variant of palivizumab confers biological activity *via* AIP2^VHH^gp130Δstalk and AIP3^VHH^gp130Δstalk

**Figure 3:**
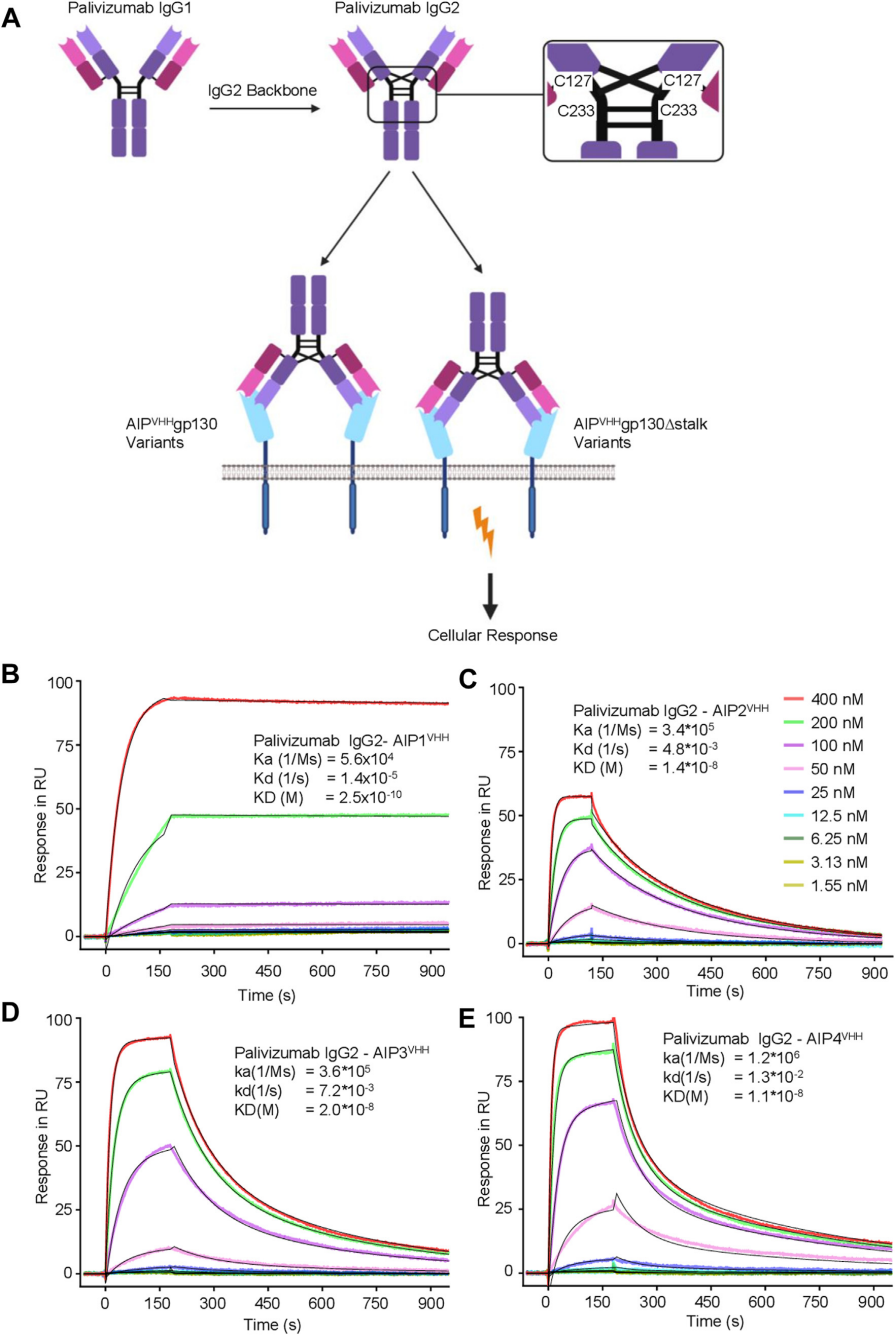
**Reformatting of palivizumab (P**^**IgG1**^**) into P**^**IgG2**^**maintained binding to AIP1**^**VHH**^**- AIP4**^**VHH**^**.***A*, schematic illustration of the transformation from P^IgG1^ to P^IgG2^ backbone and interaction with SyCyR variants. *B*–*E*, surface plasmon resonance (BIAcore) analysis of P^IgG2^ captured on a Protein A chip with soluble (*B*) AIP1^VHH^, (*C*) AIP2^VHH^, (*D*) AIP3^VHH^, and (*E*) AIP4^VHH^ as analytes (concentration range: 1.55–400 nM). Sensorgrams show response units (RU) over time (*colored lines*) with global fit (*black lines*). Analytes were injected for 120 s with 900 s dissociation time. SyCyR, synthetic cytokine receptor.

Palivizumab-induced signaling *via* AIP^VHH^gp130 was almost undetectable without secondary cross-linking (12). *In vivo*, antibody-induced cross-linking to achieve synthetic receptor activation would not be possible; therefore, we investigated whether an alternative Ig-backbone might be better suited for synthetic receptor activation. We decided to generate an IgG2 variant of palivizumab, P^IgG2^ (Fig. 3*A*, sequence in Fig. S7). Previously, it was shown that the presence of a unique disulfide crossover in human IgG2 variants corresponds with increased agonistic activity on natural receptors such as CD40 (23, 27). The variable region of the light and the heavy chain of palivizumab (for differentiation: P^IgG1^) was genetically fused to the IgG2 backbone (Fig. 3*A*). P^IgG2^ was expressed in Expi293F cells and purified from the cell supernatants *via* Protein A affinity chromatography (Fig. S4, *A* and *B*). Subsequently, we determined the interaction affinities of captured P^IgG2^ to soluble AIP1-4^VHH^ by surface plasmon resonance. AIP1-4^VHH^ binds P^IgG2^ with affinities of 0.25 nM, 14 nM, 20 nM, and 11 nM, respectively, which is in good agreement with those previously determined for P^IgG1^ (Fig. 3, *B*–*E*) (12), demonstrating that changing the backbone did not interfere with binding of palivizumab to the antiidiotypic nanobodies.

**Figure 4:**
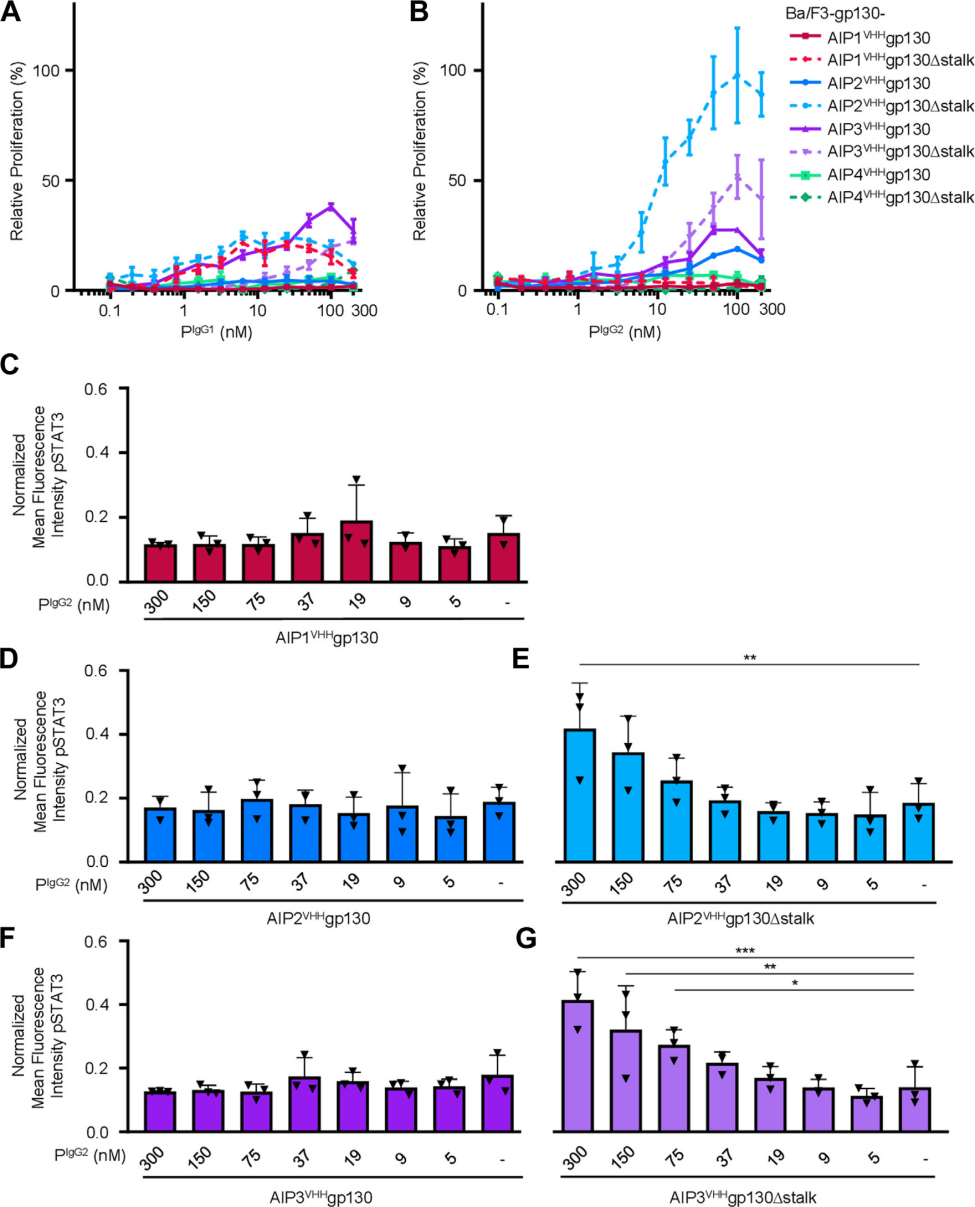
**P**^**IgG2**^**is an effectiveactivator of synthetic AIP**^**VHH**^**gp130 receptors while (P**^**IgG1**^**) is not.***A* and *B*, dose-dependent proliferation of Ba/F3-gp130 cells expressing AIP1-4^VHH^gp130 and AIP1-4^VHH^gp130Δstalk in response to (*A*) P^IgG1^ and (*B*) P^IgG2^ (0.05–300 nM). Proliferation was normalized to HIL-6 (10 ng/ml) induced proliferation for each cell line. Data represent mean ± S.D. of three biological replicates from one representative experiment out of three. *C*–*G*, normalized mean fluorescence intensity of STAT3 phosphorylation in response to P^IgG2^ (5–300 nM) for (*C*) AIP1^VHH^gp130, (*D*) AIP2^VHH^gp130, (*E*) AIP2^VHH^gp130Δstalk, (*F*) AIP3^VHH^gp130, and (*G*) AIP3^VHH^gp130Δstalk. Data represent mean ± S.D. of three biological replicates (n = 3). HIL-6, hyper-IL-6.

We compared the biological activity of P^IgG1^ and P^IgG2^ on Ba/F3-gp130 cells expressing any of the AIP1-4^VHH^gp130 or AIP1-4^VHH^gp130Δstalk variants for their ability to induce cellular proliferation and STAT3 phosphorylation (schematic Fig. 3*A*). As observed previously, P^IgG1^ induced a very weak proliferation of Ba/F3-gp130 cells expressing AIP3^VHH^gp130 but not *via* the AIP1,2,4^VHH^gp130 variants (Fig. 4*A*) (12). Interestingly, stimulation with the IgG2 subclass P^IgG2^ not only induced proliferation of Ba/F3-gp130 cells expressing AIP3^VHH^gp130 in a comparable manner as P^IgG1^, but deletion of the stalk region in AIP3^VHH^gp130Δstalk resulted in much stronger proliferation of respective Ba/F3-gp130 cells (EC50: 24.35 nM). But what appears to be of greater importance is that deletion of the stalk region in AIP2^VHH^gp130Δstalk (EC50: 11.25 nM) led to a very strong proliferative response in respective Ba/F3-gp130 cells (Fig. 4*B*). This was mirrored by determination of dose-dependent ligand-induced STAT3 phosphorylation in all Ba/F3-gp130 cells expressing the AIP1-3^VHH^gp130 or AIP2-3^VHH^gp130Δstalk variants. Here, significant STAT3 phosphorylation was only achieved by stimulation of Ba/F3-gp130 cells expressing AIP2^VHH^gp130Δstalk or AIP3^VHH^gp130Δstalk with the IgG2 subclass of palivizumab (Fig. 4, *C*–*G* and Fig. S8). Interestingly, only AIP3VHHgp130Δstalk stimulated with P^IgG2^ exhibited significant activation of STAT1 signaling (Fig. S9). No STAT3 phosphorylation was, however, observed in Ba/F3-gp130 cells expressing AIP1^VHH^gp130, AIP2^VHH^gp130, or AIP3^VHH^gp130 stimulated with P^IgG2^ (Fig. 4, *C*–*G*). In conclusion, we show that the engineered IgG2 subclass of palivizumab conferred biological activity specifically after simultaneous modification of the synthetic AIP2^VHH^gp130 receptor by deletion of the stalk region. Also, productive ligand-receptor pairs were generated in an iterative process of trial and error.

### A tetravalent IgG2 palivizumab variant mediates cellular apoptosis *via* AIP1^VHH^hFas

**Figure 5:**
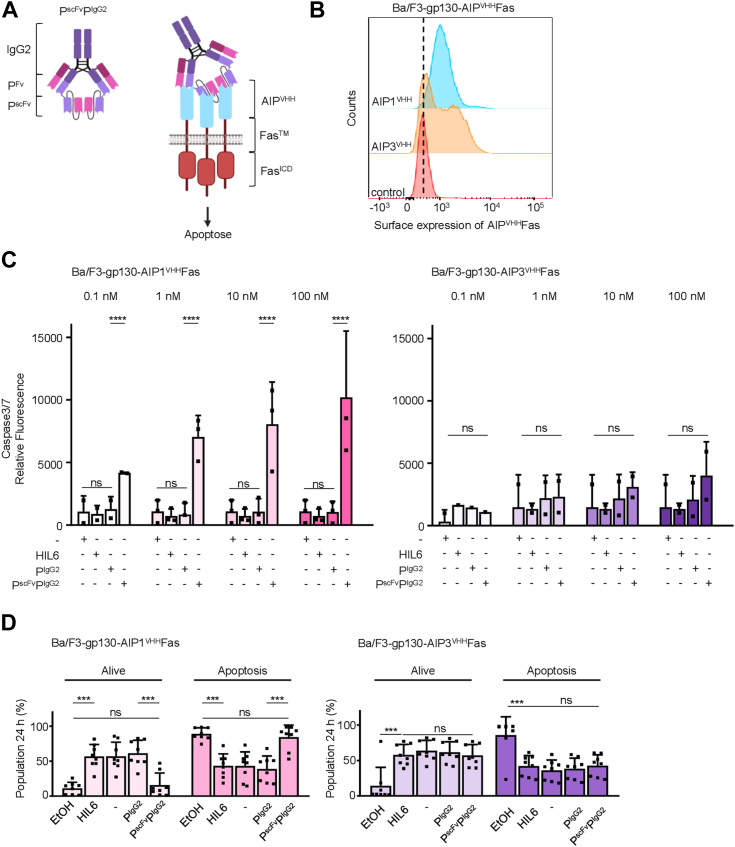
**AIP^VHH^ fusion to hFas efficiently induce cellular apoptosis**. *A*, scheme of tetravalent ligand on the P^IgG2^ backbone and its activation of trimeric Fas. *B*, flow cytometric analysis of myc-tagged synthetic receptors on Ba/F3-gp130 cells expressing AIP1^VHH^Fas and AIP3^VHH^Fas. *C*, caspase-3/7 activity after 6 h incubation of Ba/F3-gp130 cells expressing (*left*) AIP1^VHH^Fas or (*right*) AIP3^VHH^Fas with dimeric P^IgG2^ or tetrameric P^scFv^P^IgG2^ (0.1-100 nM). Controls: untreated or HIL-6 (10 ng/ml) treated cells. *D*, annexinV and 7-AAD staining of Ba/F3-gp130 cells expressing (*left*) AIP1^VHH^Fas or (*right*) AIP3^VHH^Fas after 24 h incubation with 100 nM P^IgG2^ or P^scFv^P^IgG2^. Controls: untreated, HIL-6 (10 ng/ml) treated, or HIL-6 treated and 70% EtOH washed cells. Analysis was performed by flow cytometry. HIL-6, hyper-IL-6; scFV, single-chain variable fragment.

To explore the potential for higher-order receptor clustering, we engineered a tetravalent synthetic ligand by adding a second P^scFv^ fragment to the heavy chain of the P^IgG2^ backbone. This modification resulted in a ligand with four binding sites for AIPs called P^scFv^P^IgG2^, potentially enabling receptor tetramerization (Fig. 5*A*, sequence in Fig. S10). P^scFv^P^IgG2^ was expressed in transiently transfected Expi293F cells and purified from the cell supernatants *via* Protein A affinity chromatography (Fig. S3, *A* and *B*). We functionally tested the tetravalent palivizumab variant on the synthetic Fas receptor, which requires at least trimerization for activation (28, 29, 30). The expression of AIP1^VHH^Fas and AIP3^VHH^Fas on the cell surface of Ba/F3-gp130 cells was confirmed by flow cytometry (Fig. 5*B*). Notably, activation of Fas-induced apoptosis occurred in a dose-dependent manner using the P^scFv^P^IgG2^ ligand but not P^IgG2^ on Ba/F3-gp130-AIP1^VHH^Fas after stimulation for 24 h, while no apoptosis was observed with Ba/F3-gp130-AIP3^VHH^Fas (Fig. 5*C*). We detected caspase 3/7 activity in Ba/F3-gp130-AIP1^VHH^Fas but not in Ba/F3-gp130-AIP3^VHH^Fas cells after stimulation with P^scFv^P^IgG2^ for 6 h. P^IgG2^ was unable to induce caspase 3/7 activity either *via* AIP1^VHH^Fas or AIP3^VHH^Fas after stimulation with P^scFv^P^IgG2^ for 6 h (Fig. 5*D*). These findings demonstrate the potential of our engineered ligand to induce higher-order receptor clustering and activating pathways requiring receptor trimerization or tetramerization.

### PIgG2-specific activation of SyCyR-modified primary T cells demonstrates therapeutic potential

**Figure 6:**
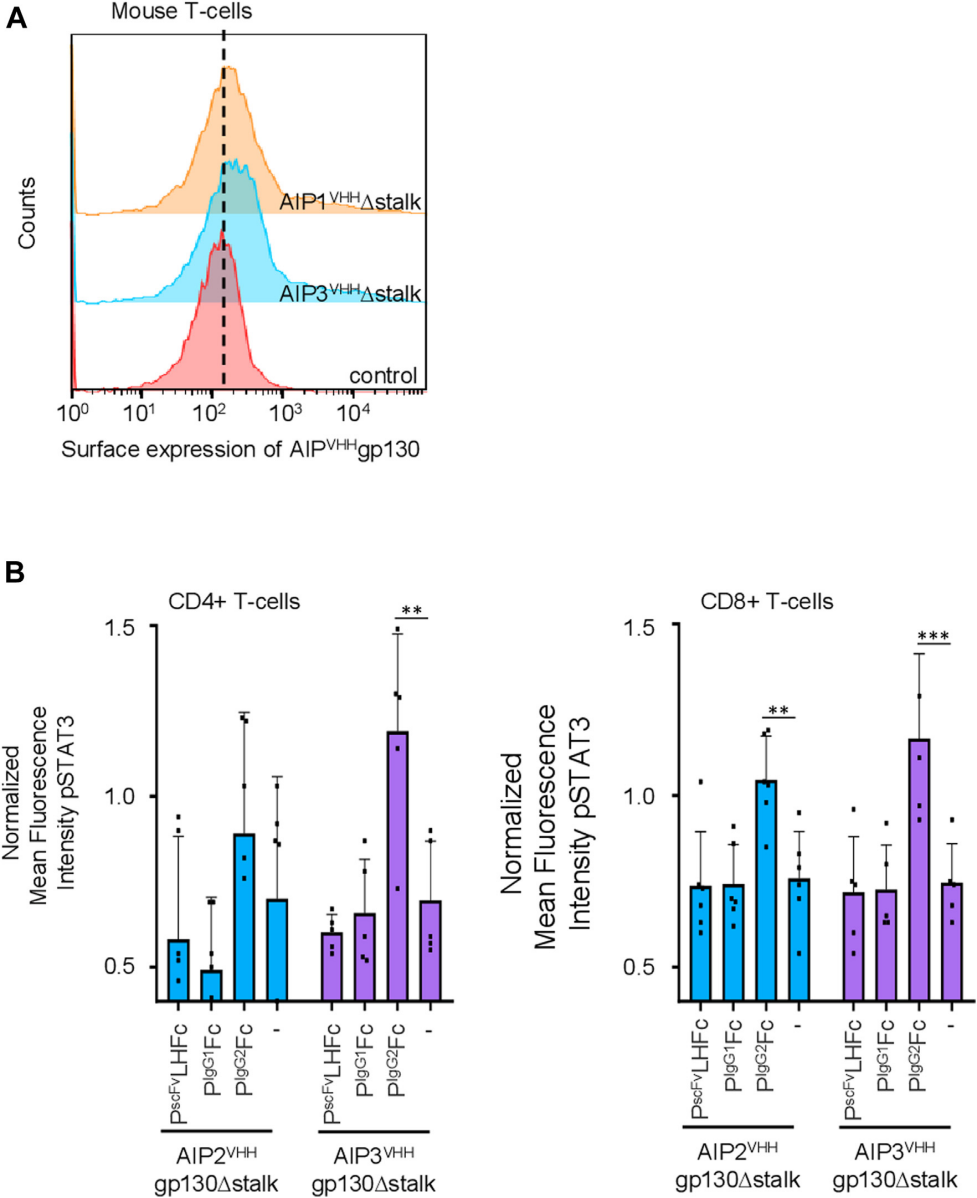
**P^IgG2^ is an effective activator of synthetic AIP^VHH^gp130Δstalk receptors on primary mouse T-Cells**. *A*, Flow cytometric analysis of myc-tagged synthetic receptors on transduced T-cells expressing AIP2^VHH^gp130Δstalk and AIP3^VHH^gp130Δstalk. *B*, Normalized mean fluorescence intensity (MFI) of STAT3 phosphorylation in response to P^scFv^LH23Fc, P^IgG1^ and P^IgG2^ (300 nM) for (*left*) AIP2^VHH^gp130Δstalk and (*right*) AIP3^VHH^gp130Δstalk. Data represent mean ± S.D. of three biological replicates (n=3).

To demonstrate the broader applicability of our system, we retrovirally transduced primary mouse T-cells to express either AIP2gp130Δstalk or AIP3gp130Δstalk SyCyRs. Flow cytometric analysis confirmed successful surface expression of both receptor variants (Fig. 6*A*). We then examined STAT3 activation in CD4+ and CD8+ T-cell populations following 45-min stimulation with 300 nM of P^scFv^LHFc, P^IgG1^, or P^IgG2^. Flow cytometric analysis revealed significant enhancement of STAT3 phosphorylation specifically in cells expressing AIP2gp130Δstalk and AIP3gp130Δstalk when treated with P^IgG2^, while P^IgG1^ and P^scFv^LHFc failed to induce comparable signaling responses (Fig. 6*B*). These findings validate the selective activation properties of our engineered receptor-ligand system in primary immune cells, highlighting its potential utility for therapeutic applications in immune cell engineering.

## Discussion

In this study, we engineered antibody-formats based on palivizumab in structure, but capable of activating a series of SyCyRs. These SyCyRs are engineered receptors designed to respond to specific synthetic ligands, allowing precise control of cellular signaling in engineered cells. The versatility of SyCyRs has been demonstrated through their successful integration with various intracellular signaling domains, including those derived from TNF, Fas, gp130, IFNAR, IL-12Rβ1, and IL-23 receptors (31, 32, 33). Our research demonstrated that the spatial configuration of receptors within dimeric complexes significantly influences signaling efficacy. We used two strategies to optimize these parameters: modifying palivizumab-derived ligand proteins with varying linker lengths and enhancing the rigidity and rotation of intracellular domains in receptor-ligand interactions.

While cytokimeras (34, 35), cytokine mimetics (36, 37), synthekines (5, 38), and fusokines (39, 40) represent innovative cytokine engineering approaches designed to activate specific cell types based on receptor expression, they cannot exclusively target engineered cells, limiting their specificity in cellular activation. In contrast, our palivizumab-based third generation SyCyR system, activated by engineered antibodies, offers unique advantages in specificity and control by exclusively targeting engineered cells. This opens up possibilities for CAR T-cell therapy, where STAT3 activation promotes effector function-related genes, and other novel gene therapies (41) Furthermore, this system could potentially function as a precise On/Off switch in CARs, enhancing control over T-cell activation different than the unspecific tyrosine kinase inhibitor dasatinib (42). Moreover, when fused to costimulatory intracellular domain such as CD28 (43) 4-1BB, or ICOS (44) it could form part of an AND-Gate logic circuit, allowing for activation only in presence of the engineered antibody (45). While the SynNotch system has been used for similar purposes, it relies on components that are not native to human cells (46). The Fas-mediated apoptosis control offers a potentially safe alternative to current suicide gene approaches like iCASP9 (47) or CD20-transduced T cells and its activator rituximab, which also targets other CD20 expressing cells (48).

The use of nanobodies in our SyCyR system offers additional advantages due to their low immunogenicity and the possibility to further humanize them (10, 49). The ability to modulate receptor proximity through antibody engineering presents new opportunities for designing therapeutic antibodies with tailored properties. For example, I-shaped antibodies, which have a more linear structure compared to traditional Y-shaped antibodies, could further enhance signaling efficacy by bringing the receptors closer together, potentially increasing the strength and duration of the signaling response (50).

Our study reveals the intricate interplay between receptor structure, ligand design, and signaling outcomes in SyCyR systems. As cell and gene therapies gain wider approval, our technology could be adapted beyond immunotherapy to fields such as regenerative medicine, metabolic disorders, or neurodegenerative diseases, leveraging its modularity and specificity for targeted cellular therapies across various medical conditions.

Deleting the stalk region in SyCyRs resulted in altered signaling responses to the originally described and only active palivizumab-variant P^scFv^LHFc, underscoring the importance of rotation, distance, and rigidity in signaling efficacy. This is important for binding of JAK kinases intracellularly in gp130 (17) and other synthetic receptors, like generalized extracellular molecule sensors (18). It is worth noting that the relative expression of receptors varies for each receptor, and STALK deletion does not affect their expression. However, receptor expression levels do not directly influence downstream signaling, likely due to signal amplification *via* STAT3. Even a small number of activated receptors can induce significant proliferation and downstream signaling. We were able to activate AIP2^VHH^gp130Δstalk while AIP2^VHH^gp130 was previously unresponsive, even though AIP2^VHH^ has similar affinity as AIP3^VHH^. AIP3^VHH^gp130Δstalk was more responsive than AIP3^VHH^gp130 suggesting that rigidity, distance, and spatial orientation or rotation, rather than high affinity are crucial factors for enhanced signaling. The importance of rigidity and distance are underscored by P^scFv^0Fc and P^scFv^4Fc disrupting signaling in three of the eight SyCyRs while the longest linker, P^scFv^8Fc was able to activate two out of eight SyCyRs. This understanding led us to hypothesize that a more rigid backbone might increase the agonism of palivizumab. We decided to use an IgG2 backbone which was shown to be more rigid due to altered disulfate bridges in its hinge region (23). We successfully transferred the P^IgG1^ variable region to an IgG2 backbone (P^IgG2^), which maintained similar affinity to AIPs as P^IgG1^ while showing improved agonistic properties on the same SyCyRs as P^scFv^8Fc.

To induce higher-order receptor clustering, we engineered a tetravalent palivizumab-variant by adding a second P^scFv^ fragment to the P^IgG2^ heavy chain resulting in P^scFv^P^IgG2^. This design was tested on the synthetically engineered Fas receptor fused to AIP1^VHH^ and AIP3^VHH^, which signals as a trimer (32), successfully activating Fas-induced apoptosis in combination with AIP1^VHH^Fas, similarly to our previous results using P^scFv^P^scFv^Fc (12) while the dimeric IgG2 variant was unable to do so. Our findings demonstrate the potential of engineered ligands to induce higher-order receptor clustering and activate pathways requiring receptor trimerization or tetramerization.

The demonstration of PIgG2-mediated activation in primary mouse T-cells expressing AIP2gp130Δstalk and AIP3gp130Δstalk SyCyRs provides critical validation of this system beyond established cell lines. The selective STAT3 phosphorylation observed in CD4+ and CD8+ T-cells specifically in response to P^IgG2^, but not P^IgG1^ or P^scFv^LHFc, confirms the engineered specificity and functionality of our system in therapeutically relevant primary immune cells.

The agonistic behavior of IgG2-reformatted palivizumab proves that antibody engineering can be used as a tool to activate synthetic cytokine signaling pathways. There are numerous IgG2 subclass therapeutic human antibodies approved by the Food and Drug Administration, the first one being approved was the epidermal growth factor receptor binding panitumumab (2006) (51), and the latest one being nemolizumab (2024) (52), an IL-31R-alpha blocker. Since P^IgG1^ is already Food and Drug Administration-approved, alterations in the backbone could potentially qualify for fast-track approval, although rigorous safety and efficacy testing would still be required but advanced technologies like organ or lab-on-a-chip systems could expedite the process (53).

## Experimental procedures

### Cloning of synthetic cytokine ligands and receptors

The pcDNA3.1 plasmid encoding P^scFv^LHFc has been previously described (12). We removed the tobacco etch virus proteolytic cleavage site by restriction digest, resulting in the plasmid encoding P^scFv^0Fc. To create variants with linkers of different lengths, we used complementary oligonucleotide hybridization. For this purpose, phosphorylated oligonucleotides (5′-GGCCGCAGAAGCAGCTGCAAAAGAAGCAGCTGCAAAAGAAGCAGCTGCAAAAGAAGCAGCTGCAAAAGC-3′ and 5′-GGCCGCTTTTGCAGCTGCTTCTTTTGCAGCTGCTTCTTTTGCAGCTGCTTCTTTTGCAGCTGCTTCTGC-3′ or 5′-GGCCGCTGAGGCCGCTGCTAAGGAGGCCGCCGCTAAGGAGGCTGCCGCCAAGGAGGCCGCTGCCAAGGAGGCTGCTGCCAAGGAAGCCGCCGCCAAGGAAGCTGCCGCCAAAGAGGCCGCCGCCAAAGC-3′ and 5′-GGCCGCTTTGGCGGCGGCCTCTTTGGCGGCAGCTTCCTTGGCGGCGGCTTCCTTGGCAGCAGCCTCCTTGGCAGCGGCCTCCTTGGCGGCAGCCTCCTTAGCGGCGGCCTCCTTAGCAGCGGCCTCAGC-3′ for P^scFv^4Fc and P^scFv^8Fc, respectively) were resuspended in annealing buffer (10 mM Tris, pH 7.5–8.0, 50 mM NaCl, and 1 mM EDTA) and mixed in equimolar concentrations. The designed primers incorporated NotI overhangs, facilitating subsequent cloning into the hinge region before the Fc tag. This resulted in the pcDNA3.1-Fc vector coding for an N-terminal signal peptide and Myc tag (EQKLISEEDL), and a C-terminal linker of either four or eight EAAAK repetitions followed by a human IgG1-Fc tag, resulting in P^scFv^4Fc and P^scFv^8Fc, respectively. For the IgG2 backbone, we obtained primary sequences of IgG2 C232SκC214S described by Orr *et al.* (23). These sequences were reverse translated and codon optimized. The variable region of palivizumab, derived from the US patent (US6955717B2), was incorporated into the light and heavy constant chains. These chains were separated by an F2A peptide sequence (54) to ensure efficient expression of both chains from a single construct. The complementary DNA was synthesized by BioCat (Heidelberg, Germany). To generate stalk variants of the SyCyRs, we performed deletion PCR using the primers (Primer forward: 5′-GCCATCGTGGTGCCT-3′ Primer reverse: 5′-GAATTTAGGGGTGGTGAAGGT-3′) This PCR strategy resulted in pMOWS-AIP1-4^VHH^gp130Δstalk variants.

### Cells and reagents

The generation of Ba/F3-gp130 cells was described elsewhere (55). The packaging cell line Phoenix-Eco was received from Ursula Klingmüller (DKFZ). Cell lines were grown in Dulbecco's modified Eagle's medium high glucose culture medium (GIBCO, Life Technologies) supplemented with 10% fetal bovine serum (GIBCO, Life Technologies), 60 mg/l penicillin, and 100 mg/l streptomycin (Genaxxon bioscience GmbH) at 37 °C with 5% CO_2_. Proliferation of Ba/F3-gp130 cells was maintained in the presence of 0.2% (10 ng/ml) human HIL-6 (24). Expi-293F cells (Thermo Fisher Scientific) were cultured in Expi293 expression medium without antibiotics until they reached a density of 3 to 5 × 10^6^ cells/ml in a 37 °C incubator with 8% CO_2_ on an orbital shaker at 125 rpm. Phospho-STAT3 (Tyr705) (#557814), STAT3 (#560391), Phospho-STAT1 (Tyr701) (#612596), STAT1 (#558537), CD28: (#553294) and CD4 antibody (#558107) were obtained from BD Biosciences. Myc (71D10; cat. #2278) antibodies were obtained from Cell Signaling Technology. Rabbit anti-human IgG Fc (#31423) was obtained from Pierce and CD3e antibody was obtained from Invitrogen (#16-0031-86) (Thermo Fisher Scientific). Alexa Fluor 488–conjugated Fab goat anti-rabbit IgG (1:500, cat. #4412) was obtained from Cell Signaling Technology. CD8a antibody was obtained from BioLegend (#100766). rhIL2 (#130-097-745) and Mouse Pan T-cell Isolation Kit (#130-095-130) were obtained from Miltenyi Biotec. Palivizumab antibody was from Synagis.

### T-cell isolation from mice

Primary CD8 and CD4 T cells were isolated from the spleen and lymph node tissues of C57BL/6 mice according to Miltenyi Biotec Pan T-Cell Isolation Kit manufacturer protocol. T cells were activated in the presence of 4 μg/ml anti-CD3 (plate-bound), 1 μg/ml soluble anti-CD28, and 10 U/ml recombinant human IL-2 (rhIL-2) in the T-cell complete medium (RPMI 1640, 10% fetal bovine serum, L-glutamine-penicillin-streptomycin, and 50 μM β-mercaptoethanol). The next day, five million activated T cells were transduced with ecotropic retrovirus in the presence of 8 μg/ml polybrene and 100 U/ml rhIL-2 at 2000 g/min, 37 °C for 1 h. Afterward cells were resuspended in new T-cell medium in the presence of 100 U/ml rhIL-2 for one additional day.

### Transfection of cells

Ba/F3-gp130 cells were retrovirally transduced with the pMOWS expression plasmids coding for AIP1-4^VHH^gp130 and AIP1-4^VHH^gp130Δstalk variants as described in (32). Transduced cells were grown in Dulbecco's modified Eagle's medium as described above supplemented with 10 ng/ml HIL-6. Selection of transduced Ba/F3-gp130 cells was performed with puromycin (1.5 μg/ml) (Carl Roth) for at least two weeks. Afterward, the generated Ba/F3-gp130 cell lines were analyzed for synthetic receptor cell surface expression *via* flow cytometry.

### Mammalian expression and purification of recombinant proteins

pcDNA3.1 encoding, P^scFv^LHFc, P^scFv^LH0Fc, P^scFv^LH4Fc, P^scFv^LH8Fc, P^IgG2^, and P^scFv^P^IgG2^ were transfected into Expi-293F cells using ExpiFectamine. Reaching 4.5 to 5.5 × 10^6^ c/ml, the cells were diluted to a final density of 3 × 10^6^c/ml in 30 ml Expi293 expression medium. 30 μg of the plasmid expression vectors were used for transfection according to the manufacturer’s instructions. After 6 days, the culture was harvested by centrifugation at 450*g* at 4 °C for 5 min, followed by centrifugation of the resulting supernatant at 4000*g* at 4 °C for 20 min. The supernatant of the second centrifugation step was filtered (0.45 μm, Carl Roth cat. #P667.1) and purified by affinity chromatography. Recombinant proteins containing an Fc-tag were purified using Protein A resin (1 ml, HiTrap MabSelect PrismA) at a flow rate of 1 ml/min. The column was washed with 30 column volumes of PBS. Proteins were eluted at pH 3.2 to 3.5 using a 50 mM citric acid buffer. Fractions containing the protein peak were pooled, and the pH was adjusted to pH 7 with 1 M Tris, pH 11. Recombinant proteins containing a C-terminal Twin-Strep-tag were purified using Strep-Tactin resin (IBA cat. #2–5025–001) according to the manufacturer’s instructions. All purified proteins were rebuffered to PBS using illustra NAP25 columns (GE HealthCare Life Sciences). Protein concentrations were determined by measuring absorbance at 280 nm, and samples were flash-frozen in liquid nitrogen. Protein quality was assessed by SDS-PAGE, Coomassie brilliant blue staining and functional testing. We obtained 10.5 mg from 90 ml P^scFv^LHFc, 2.6 mg from 60 ml P^scFv^0LHFc, 8 mg from 90 ml P^scFv^4LHFc, 1 mg from 60 ml P^scFv^8LHFc, 7 mg from 90 ml P^IgG2^, and 3.5 mg from 90 ml P^scFv^P^IgG2^.

### Surface plasmon resonance

For surface plasmon resonance experiments, the Biacore X100 instrument (Cytiva Life Sciences) and Protein A sensor chip (Cytiva Life Sciences, # 29127558) were used. P^IgG2^, P^IgG2^ and P^scFv^Fc variants were captured to a single flow cell at a level of about 500 response units per cycle. Three samples containing only running buffer were injected over both ligand and reference flow cell, followed by AIP1-4^VHH^ serially diluted from 400 nM to 1.5 nM, with an independent final replicate of 12.5 nM. AIP1-4^VHH^ were injected at a flow rate of 30 μl/min for 120 s, and the dissociation was measured for 900 s. Experiments were carried out at 25 °C in PBS pH 7.4, composed of 137 mM NaCl, 2.7 mM KCl, 12 mM HPO_4_^2−^/H_2_PO_4_^−^, and 0.05% (v/v) surfactant P20 (GE HealthCare). The resulting data were reference subtracted and fit to a 1:1 binding model using the Biacore X100 Evaluation software (https://www.cytivalifesciences.com/en/us/support/software/biacore-downloads/biacore-x100-software) V 2.0.1.

### Cell surface detection of AIP1-4^VHH^gp130Δstalk *via* flow cytometry

Briefly, 5 × 10^5^ Ba/F3-gp130 cells and variants thereof were washed in fluorescence-activated cell sorting (FACS) buffer (PBS and 1% bovine serum albumin (BSA)) and then incubated in 50 μl of FACS buffer containing primary myc antibody (1:100). After incubation for 1 h at room temperature (RT), cells were washed and resuspended in 50 μl of FACS buffer containing secondary antibody (Alexa Fluor 488–conjugated Fab anti-rabbit IgG 1:500) and incubated for 1 h at RT. Cells were washed and resuspended in 500 μl of FACS buffer and analyzed by flow cytometry (BD FACSCanto II flow cytometer using the FACSDiva software, BD Biosciences, https://www.bdbiosciences.com/en-us/products/software/instrument-software/bd-facsdiva-software). Data analysis was conducted using FlowJo Version 10 (https://www.flowjo.com/solutions/flowjo/downloads) (Tree Star Inc).

### Proliferation assays

Ba/F3-gp130 cells and variants thereof were washed, and 1 × 10^4^ cells were cultured for three days in a final volume of 100 μl in the presence of (synthetic) cytokines and antibodies. The CellTiter-Blue Reagent was used to determine cellular viability by recording the fluorescence (excitation 560 nm and emission 590 nm) using an Infinite M200 PRO plate reader (Tecan) immediately after adding 20 μl of reagent per well (time point 0) and up to 120 min thereafter. All conditions were measured in triplicate per experiment. Fluorescence values were normalized by subtraction of time point 0 values. All experiments were performed at least three times, and one representative experiment was selected.

### Stimulation of cells and STAT3/pSTAT3 detection by flow cytometry

Ba/F3-gp130 cells or primary mouse T-cells and variants thereof (10^6^ cells) were washed three times with PBS and starved in serum-free medium for 3 h. Cells were then stimulated with the indicated ligands or antibodies for 45 min (or as specified) in 200 μl in a 96 well plate. Primary T-cells were washed in PBS and subsequently incubated for 15 min with myc antibody on ice. Following a PBS wash, cells were incubated for 15 min with fluorophore-conjugated antibodies against CD4 and CD8 along with Alexa Fluor 488–conjugated secondary antibody targeting the myc antibody. Stained cells were subsequently subjected to phosphorylation analysis. For phosphorylation analysis using flow cytometry, cells were centrifuged (300*g*, for 5 min), 100 μl of medium was discarded, and the remaining 100 μl of the stimulated cell suspension were mixed with 100 μl of 4% paraformaldehyde and incubated at 37 °C for 10 min. Cells were then centrifuged (300*g*, 4 °C for 5 min). The resulting cell pellets were resuspended in ice-cold 90% methanol and incubated at −20 °C for 10 min. Subsequently, cells were washed twice with ice-cold BSA-EDTA buffer (1% BSA, 2 mM EDTA in PBS). Cells were then incubated overnight with fluorophore-coupled primary antibodies against STAT1 and STAT3 (1:100 dilution) or phospho-STAT1 (Y701) and phospho-STAT3 (Y705) (1:200 dilution) 2% BSA and 0.5 mM EDTA in PBS. Prior to analysis, cells were washed three times in 1% BSA-EDTA buffer. STAT3 and STAT1 activation was calculated by taking the ratio of the mean fluorescence intensity of total STAT1 or STAT3 to phosphorylated STAT1 (pSTAT1) or phosphorylated STAT3 (pSTAT3), respectively, for each sample; these values were normalized to those obtained with HIL6. Flow cytometry analysis was performed using FACS Canto II (BD Biosciences). Data were analyzed using FlowJo software (version 10.6.1, BD Biosciences).

### AnnexinV/7-aminoactinomycin D staining

Ba/F3-gp130 cell lines were washed three times with PBS. Subsequently, 1.25 × 10^5^ cells were used per well and incubated with the indicated cytokines for 24 h. For the ethanol condition, cells were only incubated with HIL-6 (10 ng/ml). Ethanol treatment replaced the last washing step before the measurement. Cells were washed twice with ice-cold PBS and if indicated with 70% ethanol. Cells were resuspended in 300 μl Annexin V binding buffer (BD Bioscience) with 0.5 μl Annexin V-PE (ImmunoTools) and incubated for 15 min in the dark at RT. One microliter of 7-aminoactinomycin D (R&D Systems) was added before analysis was carried out by flow cytometry recording 20,000 events.

### Fluorimetric caspase 3/7 assay

Ba/F3-gp130 cells were washed three times with PBS. Subsequently, 1.25 × 10^5^ cells were incubated with the indicated cytokines for 6 h in a 96-well plate in a volume of 100 μl. Subsequently, induction of apoptosis was determined using Amplite Fluorimetric Caspase 3/7 Assay kit (AAT Bioquest, Inc) according to manufacturer’s recommendations. In brief, 100 μl of the caspase-3/7 working solution was added to the cells and incubated for 2 h at RT. After centrifugation at 450*g* for 1 min, the fluorescence (excitation 350 nm, emission 450 nm) using an Infinite M200 PRO plate reader (Tecan) was determined.

### Statistical analyses

For proliferation assays, a representative experiment of n ≥ three assays with comparable results is displayed. EC_50_ values were determined using a nonlinear regression analysis with variable slope calculation in GraphPad Prism 8.0 (version 8.0.2 for Windows, GraphPad Software, www.graphpad.com) from three individual experiments. The data are presented as means ± SD. For multiple comparisons, two-way ANOVA including Bonferroni as statistical hypothesis test was used (GraphPad Prism 8.0.2, GraphPad Software Inc.). Statistical significance was set at the level of *p* < 0.05 (∗*p* < 0.05; ∗∗*p* < 0.01; ∗∗∗*p* < 0.001).

## Data availability

All materials will be provided upon request.

## Supporting information

This article contains supporting information.

## Conflict of interest

The authors declare that they have no conflicts of interest with the contents of this article.
