# Supplementary material for: An engineered palivizumab IgG2 subclass for synthetic gp130 and fas-mediated signaling
Source: J Biol Chem. 2025 Jan 17;301(3):108205. doi: 10.1016/j.jbc.2025.108205 (PMC11872477; doi:10.1016/j.jbc.2025.108205)
Supplement: Supplemental Figures [file mmc1.pptx]

## Slide 1
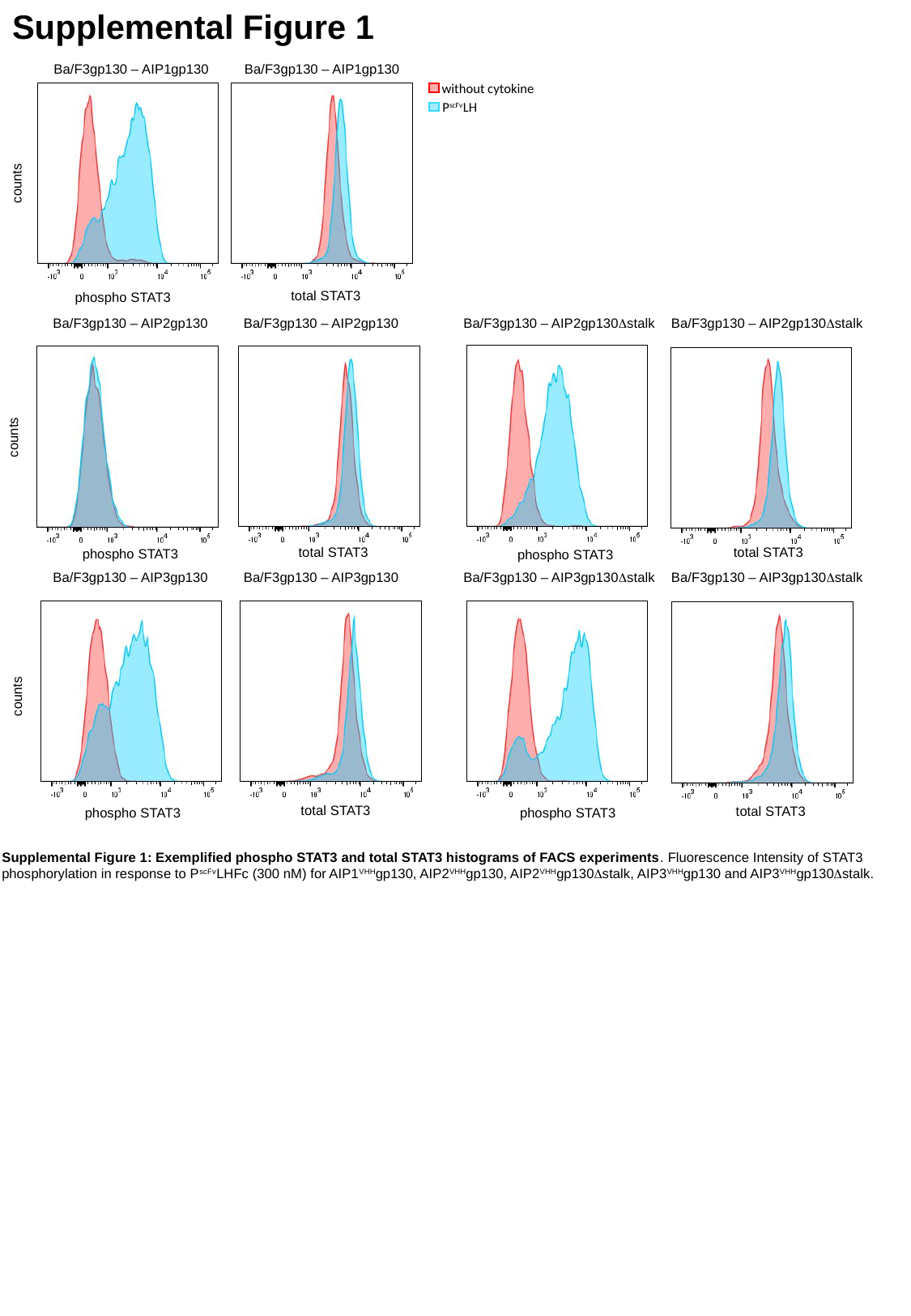

Supplemental Figure 1
Ba/F3gp130 – AIP1gp130
Ba/F3gp130 – AIP1gp130
without cytokine
PscFvLH
counts
total STAT3
phospho STAT3
Ba/F3gp130 – AIP2gp130
Ba/F3gp130 – AIP2gp130
Ba/F3gp130 – AIP2gp130Dstalk
Ba/F3gp130 – AIP2gp130Dstalk
counts
total STAT3
total STAT3
phospho STAT3
phospho STAT3
Ba/F3gp130 – AIP3gp130
Ba/F3gp130 – AIP3gp130
Ba/F3gp130 – AIP3gp130Dstalk
Ba/F3gp130 – AIP3gp130Dstalk
counts
total STAT3
total STAT3
phospho STAT3
phospho STAT3
Supplemental Figure 1: Exemplified phospho STAT3 and total STAT3 histograms of FACS experiments. Fluorescence Intensity of STAT3 phosphorylation in response to PscFvLHFc (300 nM) for AIP1VHHgp130, AIP2VHHgp130, AIP2VHHgp130Dstalk, AIP3VHHgp130 and AIP3VHHgp130Dstalk.

## Slide 2
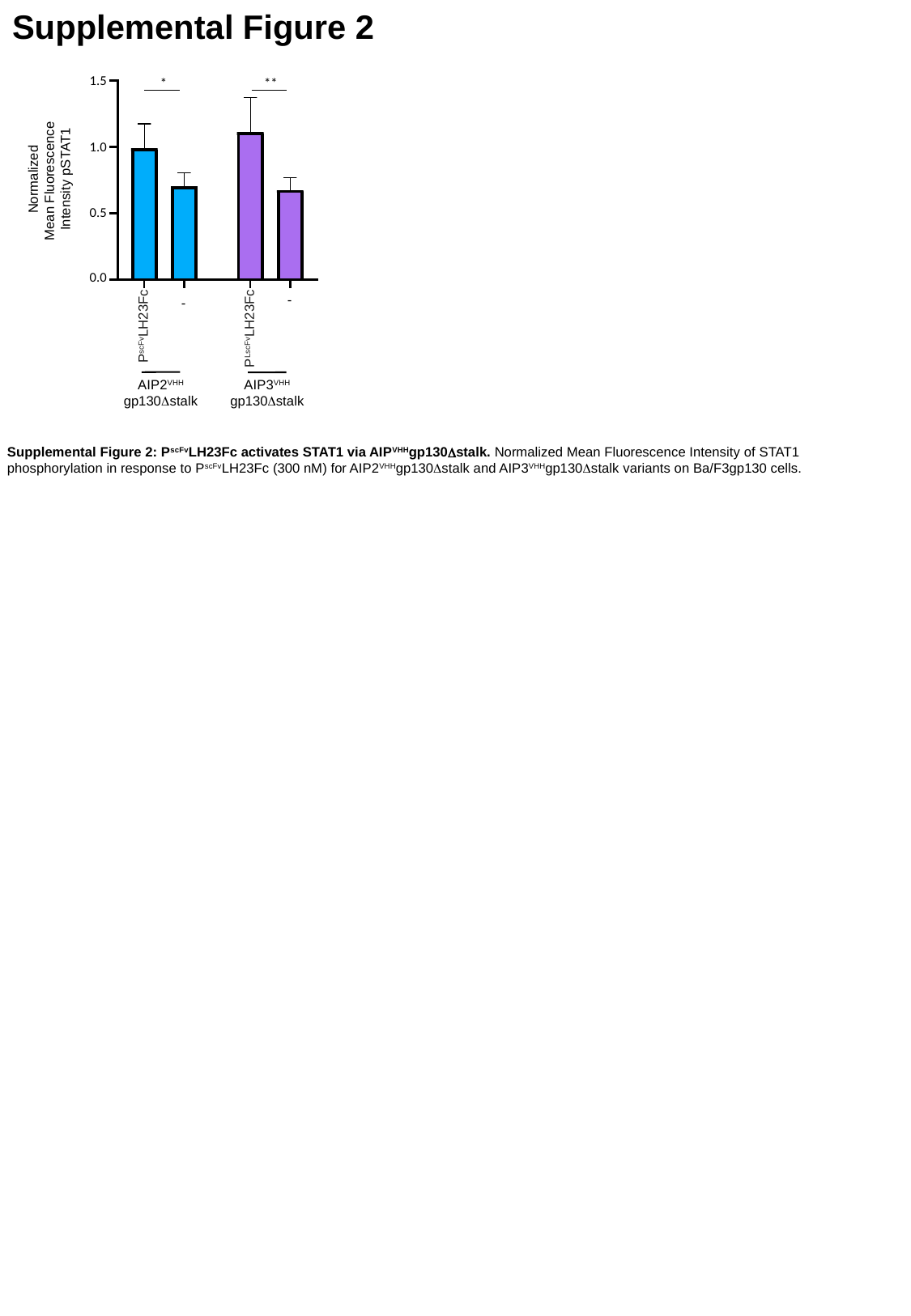

Supplemental Figure 2
1.5
*
**
1.0
Normalized
Mean Fluorescence
Intensity pSTAT1
0.5
0.0
-
PLscFvLH23Fc
-
PscFvLH23Fc
AIP3VHH
gp130Dstalk
AIP2VHH
gp130Dstalk
Supplemental Figure 2: PscFvLH23Fc activates STAT1 via AIPVHHgp130Δstalk. Normalized Mean Fluorescence Intensity of STAT1 phosphorylation in response to PscFvLH23Fc (300 nM) for AIP2VHHgp130Dstalk and AIP3VHHgp130Dstalk variants on Ba/F3gp130 cells.

## Slide 3
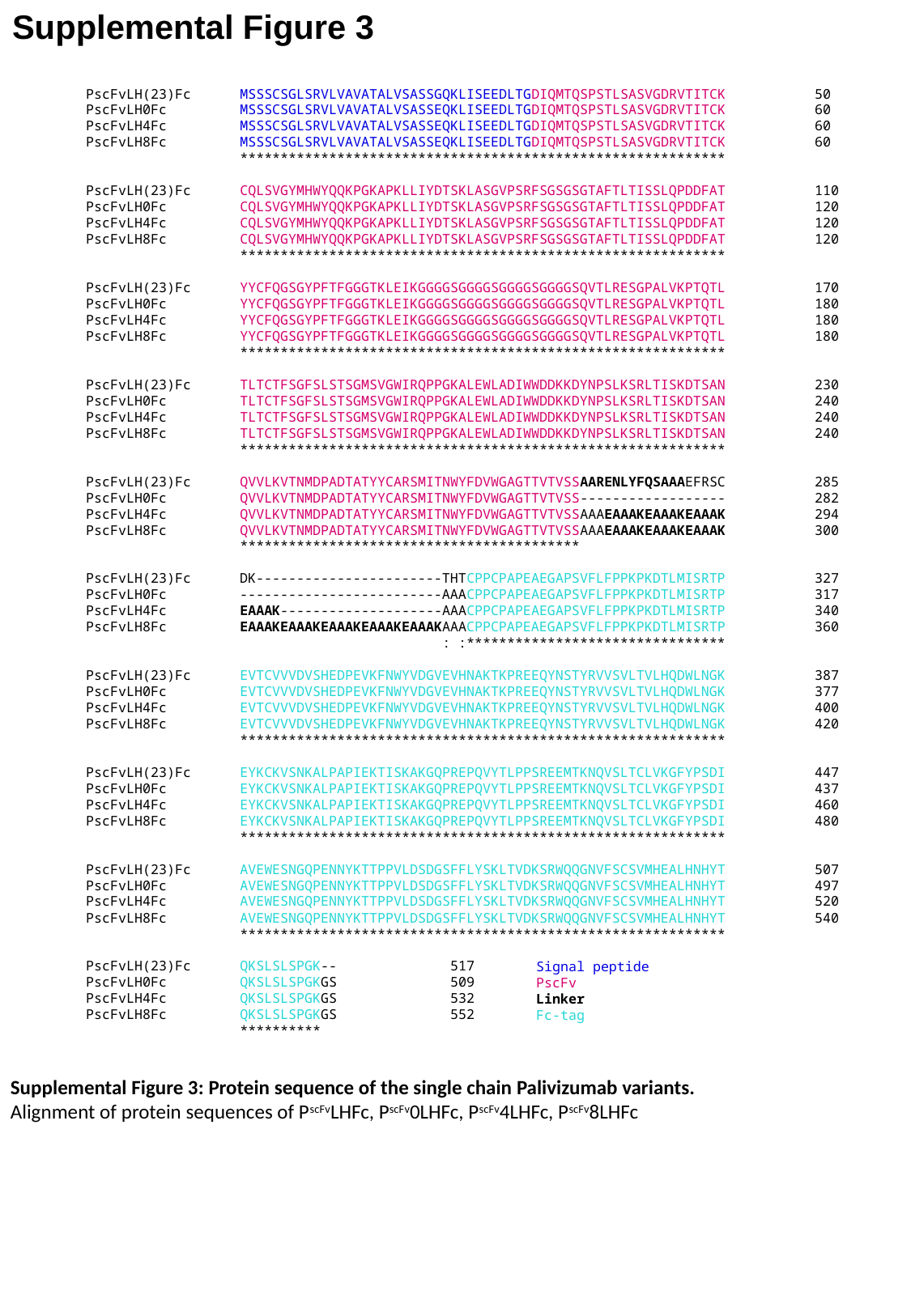

Supplemental Figure 3
PscFvLH(23)Fc MSSSCSGLSRVLVAVATALVSASSGQKLISEEDLTGDIQMTQSPSTLSASVGDRVTITCK	50
PscFvLH0Fc MSSSCSGLSRVLVAVATALVSASSEQKLISEEDLTGDIQMTQSPSTLSASVGDRVTITCK	60
PscFvLH4Fc MSSSCSGLSRVLVAVATALVSASSEQKLISEEDLTGDIQMTQSPSTLSASVGDRVTITCK	60
PscFvLH8Fc MSSSCSGLSRVLVAVATALVSASSEQKLISEEDLTGDIQMTQSPSTLSASVGDRVTITCK	60
 ************************************************************
PscFvLH(23)Fc CQLSVGYMHWYQQKPGKAPKLLIYDTSKLASGVPSRFSGSGSGTAFTLTISSLQPDDFAT	110
PscFvLH0Fc CQLSVGYMHWYQQKPGKAPKLLIYDTSKLASGVPSRFSGSGSGTAFTLTISSLQPDDFAT	120
PscFvLH4Fc CQLSVGYMHWYQQKPGKAPKLLIYDTSKLASGVPSRFSGSGSGTAFTLTISSLQPDDFAT	120
PscFvLH8Fc CQLSVGYMHWYQQKPGKAPKLLIYDTSKLASGVPSRFSGSGSGTAFTLTISSLQPDDFAT	120
 ************************************************************
PscFvLH(23)Fc YYCFQGSGYPFTFGGGTKLEIKGGGGSGGGGSGGGGSGGGGSQVTLRESGPALVKPTQTL	170
PscFvLH0Fc YYCFQGSGYPFTFGGGTKLEIKGGGGSGGGGSGGGGSGGGGSQVTLRESGPALVKPTQTL	180
PscFvLH4Fc YYCFQGSGYPFTFGGGTKLEIKGGGGSGGGGSGGGGSGGGGSQVTLRESGPALVKPTQTL	180
PscFvLH8Fc YYCFQGSGYPFTFGGGTKLEIKGGGGSGGGGSGGGGSGGGGSQVTLRESGPALVKPTQTL	180
 ************************************************************
PscFvLH(23)Fc TLTCTFSGFSLSTSGMSVGWIRQPPGKALEWLADIWWDDKKDYNPSLKSRLTISKDTSAN	230
PscFvLH0Fc TLTCTFSGFSLSTSGMSVGWIRQPPGKALEWLADIWWDDKKDYNPSLKSRLTISKDTSAN	240
PscFvLH4Fc TLTCTFSGFSLSTSGMSVGWIRQPPGKALEWLADIWWDDKKDYNPSLKSRLTISKDTSAN	240
PscFvLH8Fc TLTCTFSGFSLSTSGMSVGWIRQPPGKALEWLADIWWDDKKDYNPSLKSRLTISKDTSAN	240
 ************************************************************
PscFvLH(23)Fc QVVLKVTNMDPADTATYYCARSMITNWYFDVWGAGTTVTVSSAARENLYFQSAAAEFRSC	285
PscFvLH0Fc QVVLKVTNMDPADTATYYCARSMITNWYFDVWGAGTTVTVSS------------------	282
PscFvLH4Fc QVVLKVTNMDPADTATYYCARSMITNWYFDVWGAGTTVTVSSAAAEAAAKEAAAKEAAAK	294
PscFvLH8Fc QVVLKVTNMDPADTATYYCARSMITNWYFDVWGAGTTVTVSSAAAEAAAKEAAAKEAAAK	300
 ******************************************
PscFvLH(23)Fc DK-----------------------THTCPPCPAPEAEGAPSVFLFPPKPKDTLMISRTP	327
PscFvLH0Fc -------------------------AAACPPCPAPEAEGAPSVFLFPPKPKDTLMISRTP	317
PscFvLH4Fc EAAAK--------------------AAACPPCPAPEAEGAPSVFLFPPKPKDTLMISRTP	340
PscFvLH8Fc EAAAKEAAAKEAAAKEAAAKEAAAKAAACPPCPAPEAEGAPSVFLFPPKPKDTLMISRTP	360
 : :********************************
PscFvLH(23)Fc EVTCVVVDVSHEDPEVKFNWYVDGVEVHNAKTKPREEQYNSTYRVVSVLTVLHQDWLNGK	387
PscFvLH0Fc EVTCVVVDVSHEDPEVKFNWYVDGVEVHNAKTKPREEQYNSTYRVVSVLTVLHQDWLNGK	377
PscFvLH4Fc EVTCVVVDVSHEDPEVKFNWYVDGVEVHNAKTKPREEQYNSTYRVVSVLTVLHQDWLNGK	400
PscFvLH8Fc EVTCVVVDVSHEDPEVKFNWYVDGVEVHNAKTKPREEQYNSTYRVVSVLTVLHQDWLNGK	420
 ************************************************************
PscFvLH(23)Fc EYKCKVSNKALPAPIEKTISKAKGQPREPQVYTLPPSREEMTKNQVSLTCLVKGFYPSDI	447
PscFvLH0Fc EYKCKVSNKALPAPIEKTISKAKGQPREPQVYTLPPSREEMTKNQVSLTCLVKGFYPSDI	437
PscFvLH4Fc EYKCKVSNKALPAPIEKTISKAKGQPREPQVYTLPPSREEMTKNQVSLTCLVKGFYPSDI	460
PscFvLH8Fc EYKCKVSNKALPAPIEKTISKAKGQPREPQVYTLPPSREEMTKNQVSLTCLVKGFYPSDI	480
 ************************************************************
PscFvLH(23)Fc AVEWESNGQPENNYKTTPPVLDSDGSFFLYSKLTVDKSRWQQGNVFSCSVMHEALHNHYT	507
PscFvLH0Fc AVEWESNGQPENNYKTTPPVLDSDGSFFLYSKLTVDKSRWQQGNVFSCSVMHEALHNHYT	497
PscFvLH4Fc AVEWESNGQPENNYKTTPPVLDSDGSFFLYSKLTVDKSRWQQGNVFSCSVMHEALHNHYT	520
PscFvLH8Fc AVEWESNGQPENNYKTTPPVLDSDGSFFLYSKLTVDKSRWQQGNVFSCSVMHEALHNHYT	540
 ************************************************************
PscFvLH(23)Fc QKSLSLSPGK--	517
PscFvLH0Fc QKSLSLSPGKGS	509
PscFvLH4Fc QKSLSLSPGKGS	532
PscFvLH8Fc QKSLSLSPGKGS	552
 **********
Signal peptide
PscFv
Linker
Fc-tag
Supplemental Figure 3: Protein sequence of the single chain Palivizumab variants. Alignment of protein sequences of PscFvLHFc, PscFv0LHFc, PscFv4LHFc, PscFv8LHFc

## Slide 4
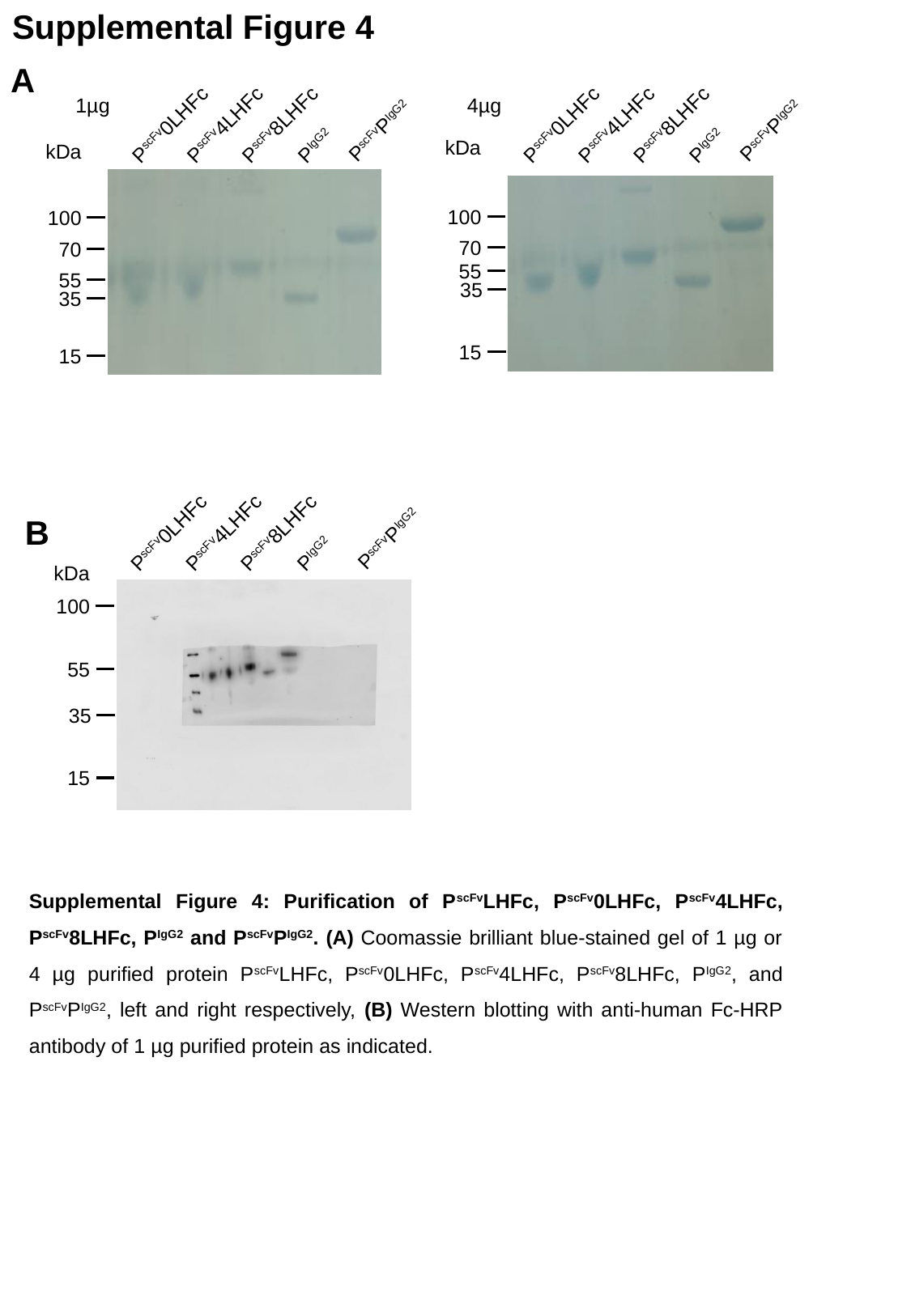

Supplemental Figure 4
A
1µg
4µg
PscFv0LHFc
PscFv4LHFc
PscFv8LHFc
PscFv0LHFc
PscFv4LHFc
PscFv8LHFc
PscFvPIgG2
PscFvPIgG2
PIgG2
PIgG2
kDa
100
70
55
35
15
kDa
100
70
55
35
15
B
PscFv0LHFc
PscFv4LHFc
PscFv8LHFc
PscFvPIgG2
PIgG2
kDa
100
55
35
15
Supplemental Figure 4: Purification of PscFvLHFc, PscFv0LHFc, PscFv4LHFc, PscFv8LHFc, PIgG2 and PscFvPIgG2. (A) Coomassie brilliant blue-stained gel of 1 µg or 4 µg purified protein PscFvLHFc, PscFv0LHFc, PscFv4LHFc, PscFv8LHFc, PIgG2, and PscFvPIgG2, left and right respectively, (B) Western blotting with anti-human Fc-HRP antibody of 1 µg purified protein as indicated.

## Slide 5
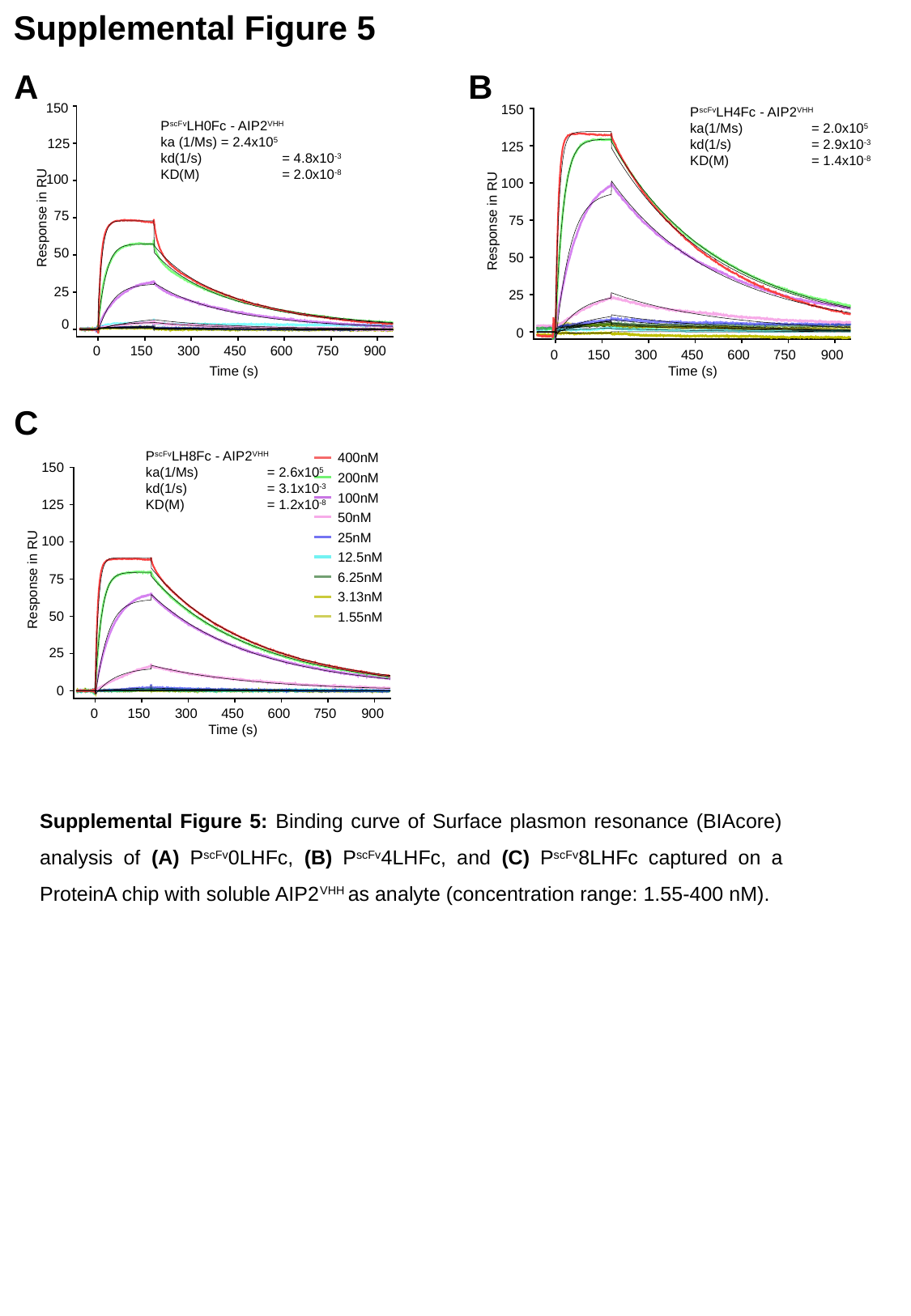

Supplemental Figure 5
A
B
150
125
100
75
50
25
0
0
150
300
450
600
750
900
Response in RU
Time (s)
150
125
100
75
50
25
0
0
150
300
450
600
750
900
Response in RU
Time (s)
PscFvLH4Fc - AIP2VHH
ka(1/Ms)	= 2.0x10^5^
kd(1/s)	= 2.9x10^-3^
KD(M)	= 1.4x10^-8^
PscFvLH0Fc - AIP2VHH
ka (1/Ms) = 2.4x10^5^
kd(1/s)	= 4.8x10^-3^
KD(M)	= 2.0x10^-8^
C
PscFvLH8Fc - AIP2VHH
ka(1/Ms)	= 2.6x10^5^
kd(1/s)	= 3.1x10^-3^
KD(M)	= 1.2x10^-8^
400nM
200nM
100nM
50nM
25nM
12.5nM
6.25nM
3.13nM
1.55nM
150
125
100
75
50
25
0
0
150
300
450
600
750
900
Response in RU
Time (s)
Supplemental Figure 5: Binding curve of Surface plasmon resonance (BIAcore) analysis of (A) PscFv0LHFc, (B) PscFv4LHFc, and (C) PscFv8LHFc captured on a ProteinA chip with soluble AIP2VHH as analyte (concentration range: 1.55-400 nM).

## Slide 6
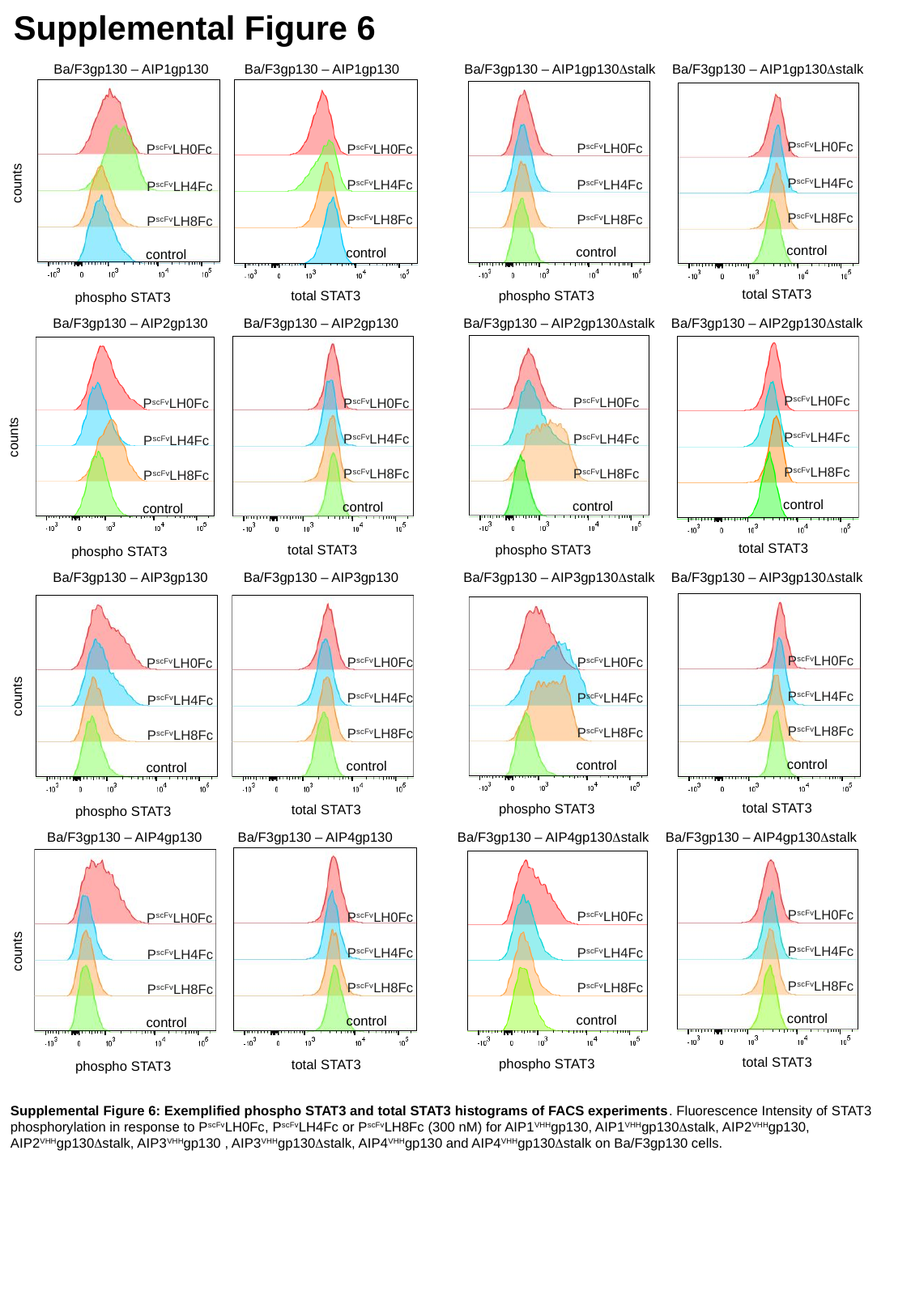

Supplemental Figure 6
Ba/F3gp130 – AIP1gp130
Ba/F3gp130 – AIP1gp130
Ba/F3gp130 – AIP1gp130Dstalk
Ba/F3gp130 – AIP1gp130Dstalk
PscFvLH0Fc
PscFvLH4Fc
PscFvLH8Fc
control
PscFvLH0Fc
PscFvLH4Fc
PscFvLH8Fc
control
total STAT3
phospho STAT3
PscFvLH0Fc
PscFvLH4Fc
PscFvLH8Fc
control
PscFvLH4Fc
PscFvLH8Fc
PscFvLH0Fc
control
total STAT3
phospho STAT3
counts
Ba/F3gp130 – AIP2gp130
Ba/F3gp130 – AIP2gp130
Ba/F3gp130 – AIP2gp130Dstalk
Ba/F3gp130 – AIP2gp130Dstalk
PscFvLH0Fc
PscFvLH4Fc
PscFvLH8Fc
control
PscFvLH0Fc
PscFvLH4Fc
PscFvLH8Fc
control
total STAT3
phospho STAT3
PscFvLH0Fc
PscFvLH4Fc
PscFvLH8Fc
control
PscFvLH4Fc
PscFvLH8Fc
PscFvLH0Fc
control
total STAT3
phospho STAT3
counts
Ba/F3gp130 – AIP3gp130
Ba/F3gp130 – AIP3gp130
Ba/F3gp130 – AIP3gp130Dstalk
Ba/F3gp130 – AIP3gp130Dstalk
PscFvLH0Fc
PscFvLH4Fc
PscFvLH8Fc
control
PscFvLH0Fc
PscFvLH4Fc
PscFvLH8Fc
control
total STAT3
phospho STAT3
PscFvLH0Fc
PscFvLH4Fc
PscFvLH8Fc
control
PscFvLH4Fc
PscFvLH8Fc
PscFvLH0Fc
control
total STAT3
phospho STAT3
counts
Ba/F3gp130 – AIP4gp130
Ba/F3gp130 – AIP4gp130
Ba/F3gp130 – AIP4gp130Dstalk
Ba/F3gp130 – AIP4gp130Dstalk
PscFvLH0Fc
PscFvLH4Fc
PscFvLH8Fc
control
PscFvLH0Fc
PscFvLH4Fc
PscFvLH8Fc
control
total STAT3
phospho STAT3
PscFvLH0Fc
PscFvLH4Fc
PscFvLH8Fc
control
PscFvLH4Fc
PscFvLH8Fc
PscFvLH0Fc
control
total STAT3
phospho STAT3
counts
Supplemental Figure 6: Exemplified phospho STAT3 and total STAT3 histograms of FACS experiments. Fluorescence Intensity of STAT3 phosphorylation in response to PscFvLH0Fc, PscFvLH4Fc or PscFvLH8Fc (300 nM) for AIP1VHHgp130, AIP1VHHgp130Dstalk, AIP2VHHgp130, AIP2VHHgp130Dstalk, AIP3VHHgp130 , AIP3VHHgp130Dstalk, AIP4VHHgp130 and AIP4VHHgp130Dstalk on Ba/F3gp130 cells.

## Slide 7
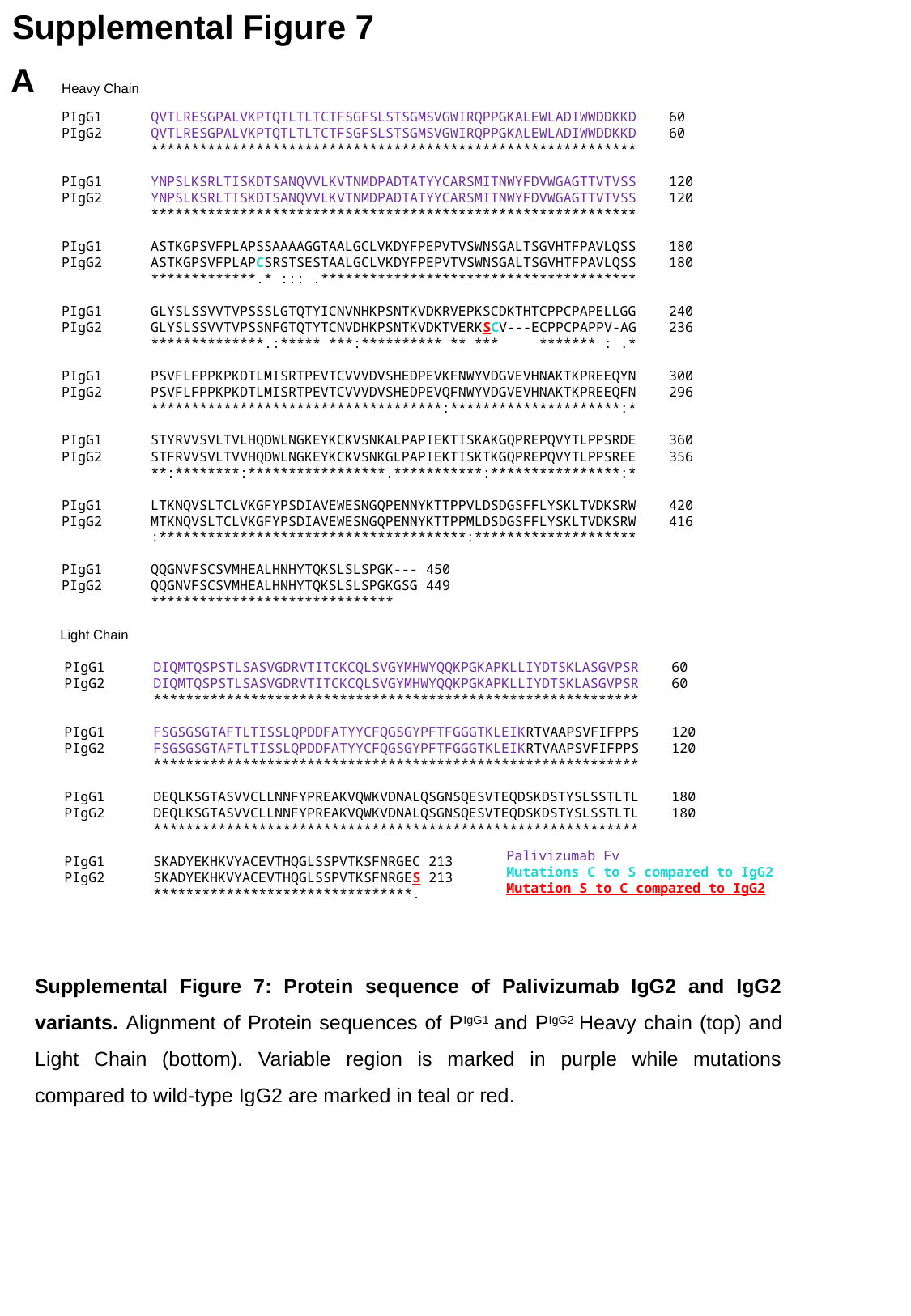

Supplemental Figure 7
A
Heavy Chain
PIgG1 QVTLRESGPALVKPTQTLTLTCTFSGFSLSTSGMSVGWIRQPPGKALEWLADIWWDDKKD	60
PIgG2 QVTLRESGPALVKPTQTLTLTCTFSGFSLSTSGMSVGWIRQPPGKALEWLADIWWDDKKD	60
 ************************************************************
PIgG1 YNPSLKSRLTISKDTSANQVVLKVTNMDPADTATYYCARSMITNWYFDVWGAGTTVTVSS	120
PIgG2 YNPSLKSRLTISKDTSANQVVLKVTNMDPADTATYYCARSMITNWYFDVWGAGTTVTVSS	120
 ************************************************************
PIgG1 ASTKGPSVFPLAPSSAAAAGGTAALGCLVKDYFPEPVTVSWNSGALTSGVHTFPAVLQSS	180
PIgG2 ASTKGPSVFPLAPCSRSTSESTAALGCLVKDYFPEPVTVSWNSGALTSGVHTFPAVLQSS	180
 *************.* ::: .***************************************
PIgG1 GLYSLSSVVTVPSSSLGTQTYICNVNHKPSNTKVDKRVEPKSCDKTHTCPPCPAPELLGG	240
PIgG2 GLYSLSSVVTVPSSNFGTQTYTCNVDHKPSNTKVDKTVERKSCV---ECPPCPAPPV-AG	236
 **************.:***** ***:********** ** *** ******* : .*
PIgG1 PSVFLFPPKPKDTLMISRTPEVTCVVVDVSHEDPEVKFNWYVDGVEVHNAKTKPREEQYN	300
PIgG2 PSVFLFPPKPKDTLMISRTPEVTCVVVDVSHEDPEVQFNWYVDGVEVHNAKTKPREEQFN	296
 ************************************:*********************:*
PIgG1 STYRVVSVLTVLHQDWLNGKEYKCKVSNKALPAPIEKTISKAKGQPREPQVYTLPPSRDE	360
PIgG2 STFRVVSVLTVVHQDWLNGKEYKCKVSNKGLPAPIEKTISKTKGQPREPQVYTLPPSREE	356
 **:********:*****************.***********:****************:*
PIgG1 LTKNQVSLTCLVKGFYPSDIAVEWESNGQPENNYKTTPPVLDSDGSFFLYSKLTVDKSRW	420
PIgG2 MTKNQVSLTCLVKGFYPSDIAVEWESNGQPENNYKTTPPMLDSDGSFFLYSKLTVDKSRW	416
 :**************************************:********************
PIgG1 QQGNVFSCSVMHEALHNHYTQKSLSLSPGK---	450
PIgG2 QQGNVFSCSVMHEALHNHYTQKSLSLSPGKGSG	449
 ******************************
Light Chain
PIgG1 DIQMTQSPSTLSASVGDRVTITCKCQLSVGYMHWYQQKPGKAPKLLIYDTSKLASGVPSR	60
PIgG2 DIQMTQSPSTLSASVGDRVTITCKCQLSVGYMHWYQQKPGKAPKLLIYDTSKLASGVPSR	60
 ************************************************************
PIgG1 FSGSGSGTAFTLTISSLQPDDFATYYCFQGSGYPFTFGGGTKLEIKRTVAAPSVFIFPPS	120
PIgG2 FSGSGSGTAFTLTISSLQPDDFATYYCFQGSGYPFTFGGGTKLEIKRTVAAPSVFIFPPS	120
 ************************************************************
PIgG1 DEQLKSGTASVVCLLNNFYPREAKVQWKVDNALQSGNSQESVTEQDSKDSTYSLSSTLTL	180
PIgG2 DEQLKSGTASVVCLLNNFYPREAKVQWKVDNALQSGNSQESVTEQDSKDSTYSLSSTLTL	180
 ************************************************************
PIgG1 SKADYEKHKVYACEVTHQGLSSPVTKSFNRGEC	213
PIgG2 SKADYEKHKVYACEVTHQGLSSPVTKSFNRGES	213
 ********************************.
Palivizumab Fv
Mutations C to S compared to IgG2
Mutation S to C compared to IgG2
Supplemental Figure 7: Protein sequence of Palivizumab IgG2 and IgG2 variants. Alignment of Protein sequences of PIgG1 and PIgG2 Heavy chain (top) and Light Chain (bottom). Variable region is marked in purple while mutations compared to wild-type IgG2 are marked in teal or red.

## Slide 8
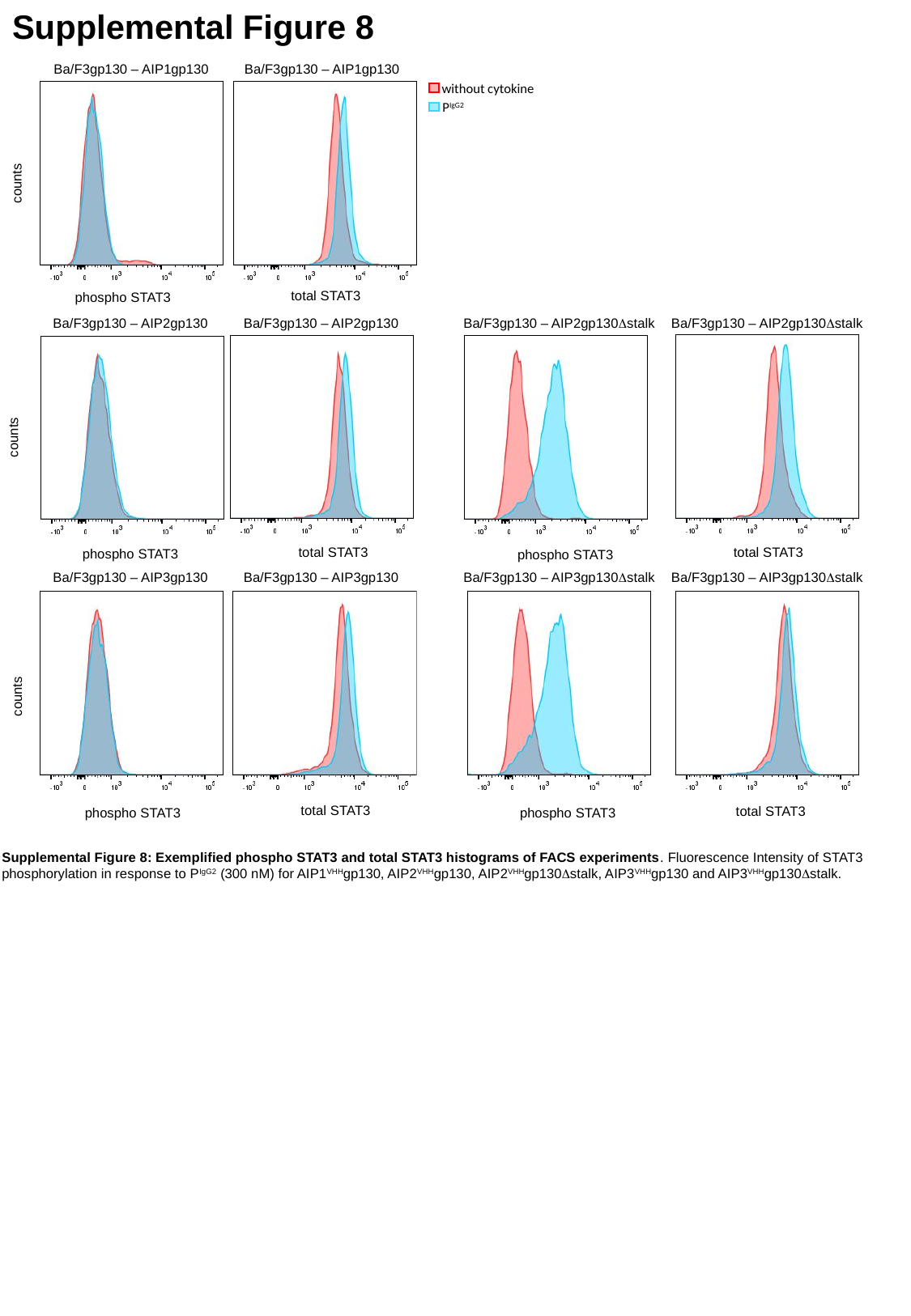

Supplemental Figure 8
Ba/F3gp130 – AIP1gp130
Ba/F3gp130 – AIP1gp130
without cytokine
PIgG2
counts
total STAT3
phospho STAT3
Ba/F3gp130 – AIP2gp130
Ba/F3gp130 – AIP2gp130
Ba/F3gp130 – AIP2gp130Dstalk
Ba/F3gp130 – AIP2gp130Dstalk
counts
total STAT3
total STAT3
phospho STAT3
phospho STAT3
Ba/F3gp130 – AIP3gp130
Ba/F3gp130 – AIP3gp130
Ba/F3gp130 – AIP3gp130Dstalk
Ba/F3gp130 – AIP3gp130Dstalk
counts
total STAT3
total STAT3
phospho STAT3
phospho STAT3
Supplemental Figure 8: Exemplified phospho STAT3 and total STAT3 histograms of FACS experiments. Fluorescence Intensity of STAT3 phosphorylation in response to PIgG2 (300 nM) for AIP1VHHgp130, AIP2VHHgp130, AIP2VHHgp130Dstalk, AIP3VHHgp130 and AIP3VHHgp130Dstalk.

## Slide 9
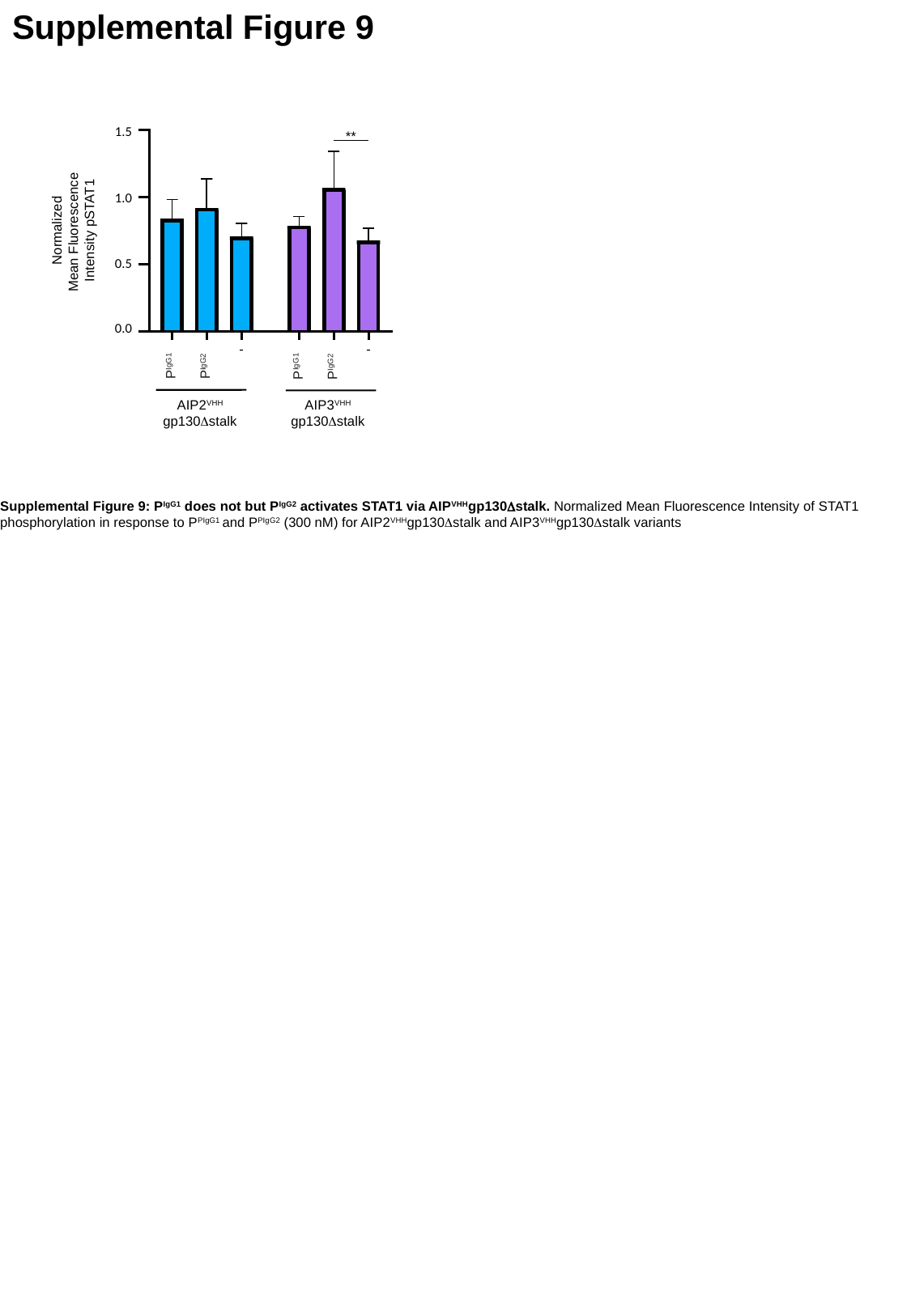

Supplemental Figure 9
1.5
1.0
0.5
0.0
**
Normalized
Mean Fluorescence
Intensity pSTAT1
-
PIgG1
PIgG2
-
PIgG1
PIgG2
AIP2VHH
gp130Dstalk
AIP3VHH
gp130Dstalk
Supplemental Figure 9: PIgG1 does not but PIgG2 activates STAT1 via AIPVHHgp130Δstalk. Normalized Mean Fluorescence Intensity of STAT1 phosphorylation in response to PPIgG1 and PPIgG2 (300 nM) for AIP2VHHgp130Dstalk and AIP3VHHgp130Dstalk variants

## Slide 10
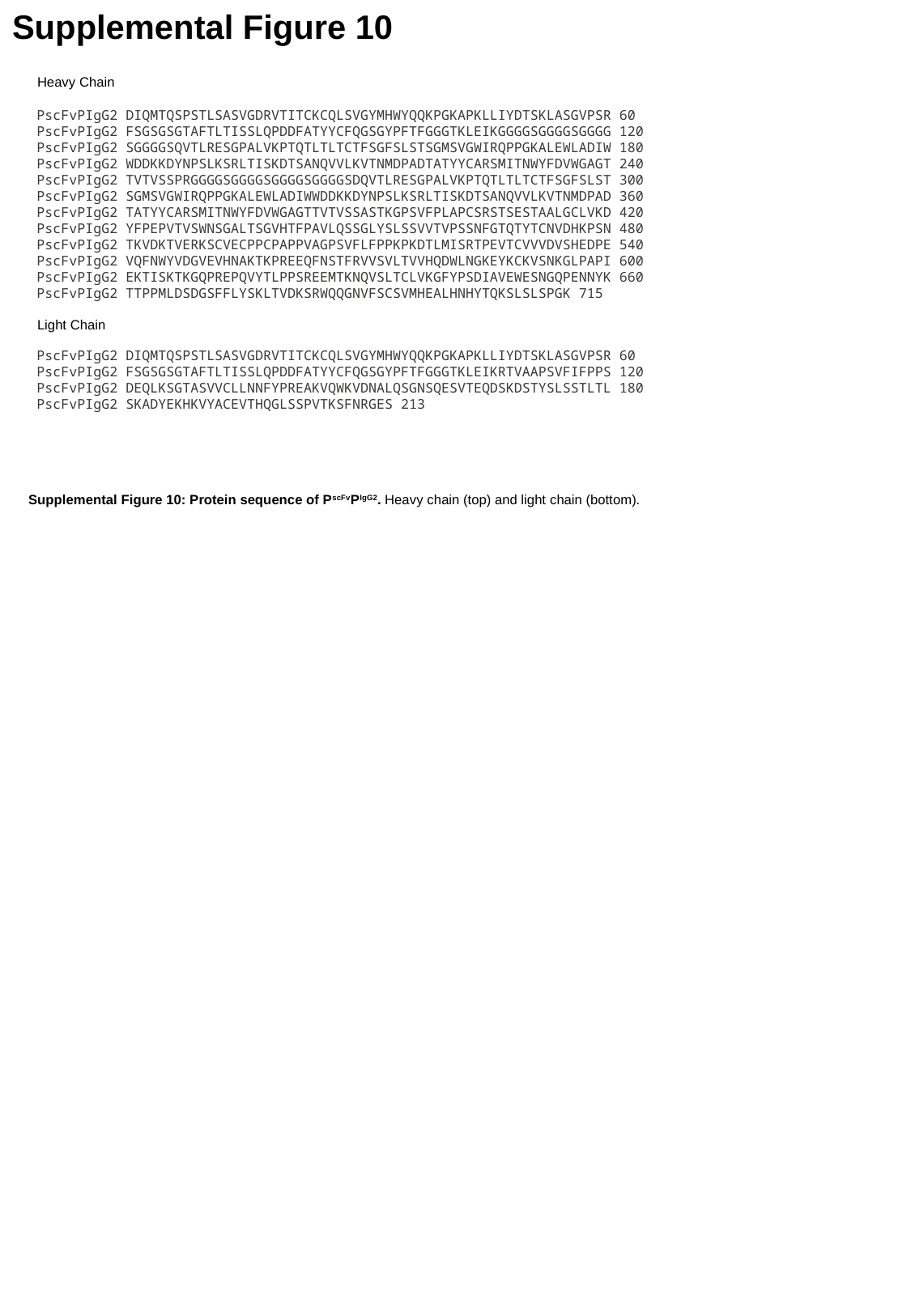

Supplemental Figure 10
Heavy Chain
PscFvPIgG2 DIQMTQSPSTLSASVGDRVTITCKCQLSVGYMHWYQQKPGKAPKLLIYDTSKLASGVPSR 60
PscFvPIgG2 FSGSGSGTAFTLTISSLQPDDFATYYCFQGSGYPFTFGGGTKLEIKGGGGSGGGGSGGGG 120
PscFvPIgG2 SGGGGSQVTLRESGPALVKPTQTLTLTCTFSGFSLSTSGMSVGWIRQPPGKALEWLADIW 180
PscFvPIgG2 WDDKKDYNPSLKSRLTISKDTSANQVVLKVTNMDPADTATYYCARSMITNWYFDVWGAGT 240
PscFvPIgG2 TVTVSSPRGGGGSGGGGSGGGGSGGGGSDQVTLRESGPALVKPTQTLTLTCTFSGFSLST 300
PscFvPIgG2 SGMSVGWIRQPPGKALEWLADIWWDDKKDYNPSLKSRLTISKDTSANQVVLKVTNMDPAD 360
PscFvPIgG2 TATYYCARSMITNWYFDVWGAGTTVTVSSASTKGPSVFPLAPCSRSTSESTAALGCLVKD 420
PscFvPIgG2 YFPEPVTVSWNSGALTSGVHTFPAVLQSSGLYSLSSVVTVPSSNFGTQTYTCNVDHKPSN 480
PscFvPIgG2 TKVDKTVERKSCVECPPCPAPPVAGPSVFLFPPKPKDTLMISRTPEVTCVVVDVSHEDPE 540
PscFvPIgG2 VQFNWYVDGVEVHNAKTKPREEQFNSTFRVVSVLTVVHQDWLNGKEYKCKVSNKGLPAPI 600
PscFvPIgG2 EKTISKTKGQPREPQVYTLPPSREEMTKNQVSLTCLVKGFYPSDIAVEWESNGQPENNYK 660
PscFvPIgG2 TTPPMLDSDGSFFLYSKLTVDKSRWQQGNVFSCSVMHEALHNHYTQKSLSLSPGK 715
Light Chain
PscFvPIgG2 DIQMTQSPSTLSASVGDRVTITCKCQLSVGYMHWYQQKPGKAPKLLIYDTSKLASGVPSR 60
PscFvPIgG2 FSGSGSGTAFTLTISSLQPDDFATYYCFQGSGYPFTFGGGTKLEIKRTVAAPSVFIFPPS 120
PscFvPIgG2 DEQLKSGTASVVCLLNNFYPREAKVQWKVDNALQSGNSQESVTEQDSKDSTYSLSSTLTL 180
PscFvPIgG2 SKADYEKHKVYACEVTHQGLSSPVTKSFNRGES 213
Supplemental Figure 10: Protein sequence of PscFvPIgG2. Heavy chain (top) and light chain (bottom).
